# Efficacy and safety of ventricular tachycardia ablation versus antiarrhythmics in ischemic cardiomyopathy: a network meta-analysis

**DOI:** 10.1007/s10840-026-02250-9

**Published:** 2026-02-20

**Authors:** Konstantinos Pamporis, Dimitrios Tsiachris, Christos-Konstantinos Antoniou, Serge Boveda, Athanasios Kordalis, Konstantinos Vlachos, Pierre Jaïs, Paschalis Karakasis, Michail Botis, Panagiotis Theofilis, Aikaterini-Eleftheria Karanikola, Konstantinos Tsioufis

**Affiliations:** 1 First Department of Cardiology, School of Medicine, Hippokration General Hospital, National and Kapodistrian University of Athens, Vasilissis Sofias 114, 11528 Athens, Greece; 2 Department of Hygiene, Social-Preventive Medicine & Medical Statistics, School of Medicine, Faculty of Health Sciences, Aristotle University of Thessaloniki, University Campus, 54124 Thessaloniki, Greece; 3 Department of Cardiology - Heart Rhythm Management, Clinique Pasteur, 45 Avenue de Lombez, 31076 Toulouse, France; 4 Vrije Universiteit Brussel (VUB), Universiteit Ziekenhuis, Laarbeeklaan 101, 1090 Jette, Brussels Belgium; 5 Electrophysiology and Heart Modeling Institute, IHU Liryc, Fondation Bordeaux Université and Université de Bordeaux-INSERM U1045, F-33600 Pessac, France; 6 Cardiac Arrhythmia Department, INSERM U1045, CHU de Bordeaux, Avenue du Haut Lévêque, F-33604 Pessac, France; 7 Electrophysiology Department, Hygeia Hospital, Erithrou Stavrou 4-5, 15123 Athens, Greece; 8 Second Cardiology Department, Hippokration General Hospital, Medical School, Aristotle University of Thessaloniki, Thessaloniki, Greece

**Keywords:** Catheter ablation, Antiarrhythmic medication, Ventricular arrhythmia, Ischemic cardiomyopathy, Network meta-analysis

## Abstract

**Background:**

Several randomized controlled trials (RCTs) have examined catheter ablation (CA) and class III antiarrhythmic drugs (AAD) in secondary prevention of ventricular arrhythmias (VA) in patients with ischemic cardiomyopathy (ICM) and an implantable cardioverter-defibrillator (ICD). This study sought to evaluate the efficacy and safety of CA versus AADs (amiodarone, sotalol) in this population.

**Methods:**

MEDLINE (Pubmed), Scopus, Cochrane and ClinicalTrials.gov were searched until October 26, 2025 for RCTs. Double-independent study selection, data extraction and quality assessment were performed. Appropriate shock and electrical storm (ES) were the primary efficacy outcomes. Risk ratios (RR) with 95% confidence intervals (CI) were calculated via random-effects frequentist models. Registered in PROSPERO: CRD42025640326.

**Results:**

Totally, 14 RCTs (13 in main analysis) and 2,237 patients (2,177 in main analysis) were analyzed. CA was superior against AAD in appropriate shocks (RR = 0.68, 95%CI = [0.47,0.99]; *p* = 0.043). A nonsignificant reduction was found in ES (RR = 0.81, 95%CI = [0.63,1.03]; *p* = 0.088) and VA recurrence (RR = 0.86, 95%CI = [0.68,1.08]; *p* = 0.197). CA was superior to AAD in heart failure (HF) exacerbation (RR = 0.76, 95%CI = [0.58,0.99], *p* = 0.043) and undetected ventricular tachycardia (VT) (RR = 0.27, 95%CI = [0.15,0.46], *p* < 0.001). No differences were noted regarding any serious adverse event, all-cause or cardiovascular mortality and cardiovascular or VA hospitalization. In secondary analyses, CA was superior against amiodarone in serious adverse events (RR = 0.33, 95%CI = [0.15,0.75]). The results remained robust in sensitivity analyses.

**Conclusions:**

CA was superior in efficacy with comparable safety compared to AAD in reducing appropriate shocks, HF and undetected VT in patients with ICM and an ICD for secondary prevention. No significant differences were found in overall VA recurrences, ES, all-cause or cardiovascular mortality.

**Graphical abstract:**

Effect estimates are expressed as risk ratios with 95% confidence intervals, while outcomes where ablation is superior are marked with asterisk (*). Abbreviations: ICD, implantable cardioverter-defibrillator; VA, ventricular arrhythmia; VT, ventricular tachycardia; CV, cardiovascular; HF, heart failure; AE, adverse events.

**Figure:**
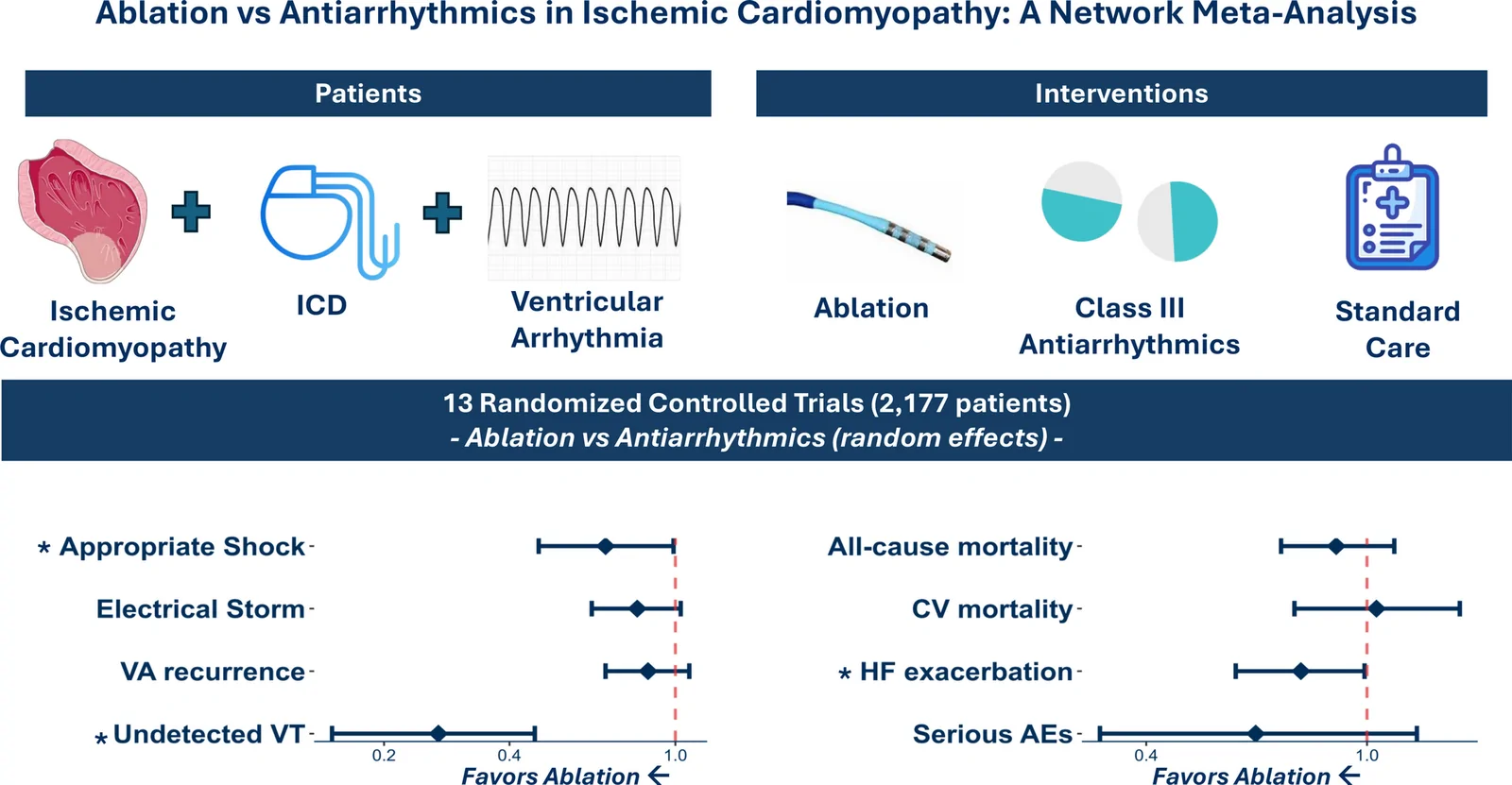

**Supplementary Information:**

The online version contains supplementary material available at https://doi.org/10.1007/s10840-026-02250-9.

## Introduction

Sudden cardiac death (SCD) attributed to lethal ventricular arrhythmias (VA) constitutes a significant cause of death in patients with coronary artery disease (CAD) and ischemic cardiomyopathy (ICM) [1]. It is estimated that approximately 50% of deaths in ICM are due to SCD [2] and CAD is the leading cause of malignant VA. Ventricular arrhythmias have also been associated with reduced quality of life and psychosocial symptoms, including depression and anxiety [3].

Based on current recommendations, patients with ICM receive implantable cardioverter defibrillators (ICD) in the context of secondary VA prevention or for primary prevention when meeting certain criteria related to New York Heart Association (NYHA) class (NYHA II or more) and a left ventricular ejection fraction (LVEF) ≤ 35% [4, 5]. Nevertheless, despite their efficacy in treating VA and reducing death [6], ICDs do not prevent VA recurrences. Hence, almost one third of patients with ICD will experience VA within a few years from implantation [7]. In this context, catheter ablation (CA) and class III antiarrhythmic drugs (AAD) constitute first-line treatments to reduce recurrences [8]. Among class III AAD, amiodarone and sotalol are currently recommended in patients with structural heart disease, with amiodarone demonstrating a superior efficacy profile [9]. Regarding CA, the strongest evidence-based recommendation is for treating VA recurrences in patients on previous amiodarone treatment, whereas both amiodarone and CA are indicated in treatment-naïve patients [4].

Previous meta-analyses have been performed, though problems with poorly defined interventions and pooling of inhomogeneous interventions in the control group (beta-blockers and AAD) [10], significantly limit their clinical applicability. Furthermore, the VANISH-2 [11] and MANTRA-VT [12] trials were recently published comparing the efficacy and safety of CA versus Class III AAD, providing evidence in favor of CA in patients with ICM. Considering the above, the present systematic review (SR) and network meta-analysis (NMA) aimed to evaluate the comparative effectiveness and safety of CA versus class III AAD in the treatment of patients with ICM and ICD for secondary prevention by synthesizing updated evidence from available RCTs.

## Methods

The present study was conducted according to the guidance of Cochrane Handbook [13] and is pre-registered in the PROSPERO database (CRD42025640326). Amendments to the original protocol (accompanied by rationale) are reported in Table S.1. Reporting was performed according to the Preferred Reporting Items for Systematic reviews and Meta-Analyses (PRISMA) 2020 statement [14] and its pertinent extension for NMA [15] (checklist in Table S.2). Ethical approval was not required due to the meta-analytical nature of the study.

### Information sources and search strategy

A systematic literature search was performed in MEDLINE (via Pubmed), Scopus and the Cochrane database from inception to October 26, 2025 (full search at Table S.3) without language restrictions. Supplemental searches were performed by citation chasing of included studies using the {citationchaser} R package and by manual search in ClinicalTrials.gov and Epistemonikos database.

### Eligibility criteria

#### Population, interventions, comparators, study design

Patients with ICM having an ICD for secondary prevention of VA were included (patients with primary prevention were also analyzed only as a sensitivity analysis). Eligible interventions were CA and class III AADs (amiodarone, sotalol) being compared against each other or against standard care (SC). Only RCTs were eligible, having enrolled ≥ 70% ICM patients with a minimum follow-up duration ≥ 6 months. The 70% cut-off was used to ensure that the pooled studies predominantly represented ICM, while avoiding unnecessary exclusion of relevant trials to maintain statistical power and generalizability. A sensitivity analysis including only studies with 100% ICM patients was also performed.

#### Outcomes

The primary efficacy outcomes were appropriate shock and electrical storm (ES). Secondary efficacy outcomes included VA recurrence, all-cause mortality, cardiovascular (CV) mortality, heart failure (HF) exacerbation, undetected VT (VT below ICD detection limit), cardiac hospitalization, VA hospitalization and appropriate antitachycardia pacing (ATP). The primary safety outcome was any serious adverse event (AE), while secondary safety outcomes included cardiac perforation, pericardial effusion (requiring drainage), stroke or transient ischemic attack (TIA), major bleeding, myocardial infarction (MI), hypothyroidism, hyperthyroidism, pulmonary toxicity and liver toxicity. Based on standardized definitions, in VA recurrence, episodes of appropriate ICD therapy (shock or ATP) or sustained (≥ 30s) VT/VF were included, while for ES, three or more VA episodes within 24h were eligible [16]. More detailed outcome definitions were adopted as perindividual study criteria. The definitions used for VA and AE in each study are presented in Table S.4, while detailed ICD programming (including zone cut-offs and treatments) in Table S.5. Data on analyzed outcomes were preferentially extracted from the intention-to-treat analyses.

### Selection process

Initially, titles and abstracts were screened by two authors independently. In this phase, studies were included for full text screening regardless of disagreements, to increase screening sensitivity. Subsequently, the same authors also independently evaluated the selected studies through full text screening. Disagreements were resolved through discussion or involvement of a third author. Abstrackr [17] and Mendeley Desktop were used.

### Data extraction

A draft extraction form was pilot-tested on three studies. After discussion and calibration exercises, a final extraction form was created. Extraction was performed independently by two authors and discrepancies were resolved through discussion or involvement of a third author. The approaches used to handle extraction issues were based on the Cochrane Handbook (Table S.6). Extracted variables are presented in Table S.7. To retrieve missing evidence, study protocols were reviewed.

### Network geometry

For the primary analysis, each network consisted of a maximum of three treatment nodes: CA, AAD and SC. Amiodarone and sotalol were merged under the category AAD. Standard care was considered the standard optimal medical treatment for patients with ICM, including revascularization, antiplatelets, hypolipidemic medication and HF treatment when indicated. As a secondary analysis, the node AAD was split into amiodarone and sotalol to further examine the comparative effectiveness and safety of interventions. This was feasible after a recent report from the VANISH-2 study [18] that reported outcomes separately for amiodarone and sotalol and under the reasonable assumption that both the SURVIVE-VT [19] (amiodarone 86%/sotalol 14%) and MANTRA-VT [12] trials (amiodarone 93%/sotalol 7%) were analyzed as amiodarone-only studies. This ancillary analysis was performed to provide further insights into the comparative effectiveness of CA against different AAD and to aid in the interpretation of the main analysis.

### Risk of bias assessments

Two authors independently assessed the risk of bias (RoB) of included studies (at the study level) using all the prespecified domains of the revised version of the Cochrane Collaboration tool (RoB 2) [20]. Disagreements were resolved upon discussion.

### Data analysis

All analyses were performed using R Statistical Software (v. 4.4.2) and the package ‘netmeta’ [21]. Categorical variables are presented as frequencies with percentages (%), while continuous variables are presented as mean with standard deviation (SD) when normally distributed, otherwise as median with interquartile range (IQR). To estimate differences between interventions, the risk ratio (RR) was used. Direct effect estimates and 95% confidence intervals (CI) were calculated using the inverse variance weighting method for random-effects models. Direct and indirect estimates were combined using random-effects frequentist NMA models with the Restricted Maximum Likelihood (REML) estimator [22]. In case of zero events, a continuity correction of 0.5 was applied. Global network homogeneity was assessed with the Cochran’s Q test and between-study variance with the τ^2^. The I^2^ metric was used to estimate between-study heterogeneity. Values of I^2^ range between 0–100, with higher values indicating larger heterogeneity. Proposed interpretation cut-offs [13] are 0%-40%: might not be important; 30%-60%: may represent moderate heterogeneity; 50%-90%: may represent substantial heterogeneity; 75%-100%: considerable heterogeneity. A common heterogeneity measure was assumed for the whole network.

The transitivity assumption was evaluated qualitatively through the comparison of the distribution of potential effect modifiers across different treatments and comparisons (age, male percentage, percentage of patients with CAD, percentage of NYHA class I, LVEF, beta blocker usage). Statistical inconsistency was assessed locally through the node-splitting method [23] and globally through the design-by-treatment test [24]. The relative contribution of the direct and indirect effects on the overall network estimate for each outcome is presented with contribution plots. To rank interventions, the P-score was used [25], which takes values between 0–1 and reflects the certainty that a given intervention is superior to competing interventions, based on the estimates and standard errors of all pairwise comparisons in the network. A higher P-score indicates a higher probability that an intervention is among the most effective options, whereas lower values indicate poorer relative performance. Small study effect (including publication bias) was assessed visually with comparison-adjusted funnel plots and formally tested with the Egger’s test. For all statistical tests, a two-tailed *p*-value < 0.05 was considered significant.

Sensitivity analyses were performed for all outcomes based on: (i) the type of VA prevention (including both primary and secondary prevention patients), (ii) the percentage of ICM (including only studies with 100% ICM patients), (iii) the number of patients (including studies that enrolled ≥ 100 patients), iv) the implementation of HF treatment (including studies that started to enroll patients after 2000, where mineralocorticoid receptor antagonists had been introduced in HF management) and v) the implementation of less aggressive ICD therapies (including studies that started to enroll after the publication of MADIT-RIT trial [26]). Additional analyses including only patients with more pronounced systolic dysfunction (e.g. LVEF < 30%) or patients presenting with ES were not feasible due to incomplete reporting in analyzed trials.

## Results

### Study selection and characteristics

**Fig. 1:**
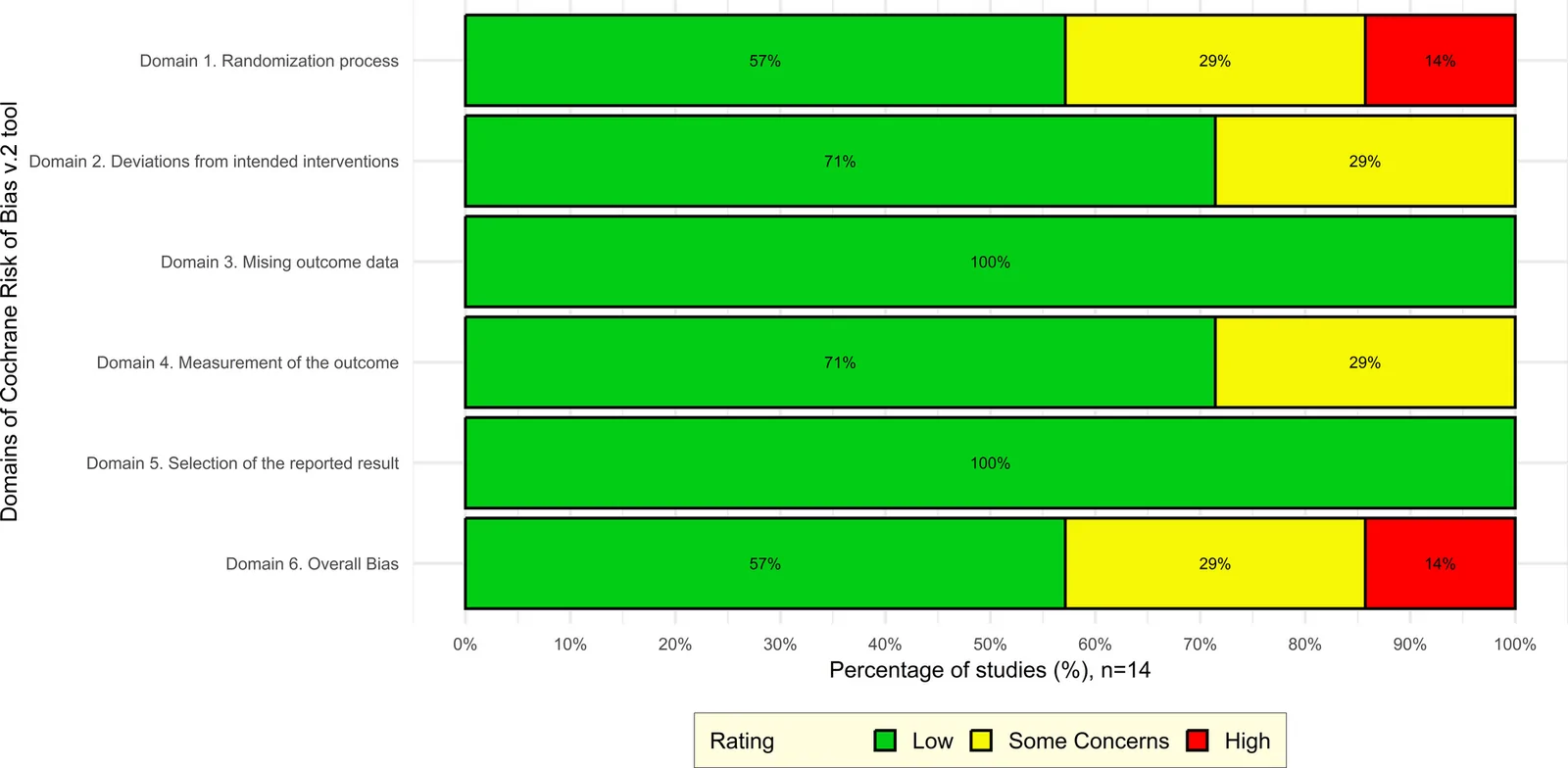
Methodological quality assessment of included primary studies based on the Cochrane Risk of Bias 2 (RoB 2) tool

In total, 10259 records were screened, 10213 articles were excluded, and 46 articles were evaluated through full text screening. After the exclusion process (excluded studies with rationale in Table S.8), 14 RCTs (13 in the main analysis for secondary prevention only; 14 in sensitivity analysis for both primary and secondary prevention) and 2237 patients (2177 in main analysis) were analyzed (Figure S.1). Study characteristics and references of included RCTs are presented in Table 1, population characteristics in Table S.9 and the procedural characteristics of each CA study (including arrhythmia-related inclusion criteria) in Table S.10. The distribution of potential effect modifiers across different treatment nodes stratified by each outcome is presented in Table S.11 and across different comparisons in Table S.12. The mean follow-up time was 24 (11) months, the mean age was 65.5 (3.2) years, the mean LVEF was 34.3% (2.8), the mean use of beta-blockers was 83%, 95% of patients had ICM, and publication year ranged between 1998–2025. Ten out of 14 (71%) studies used CA, 4/14 (29%) used amiodarone only, 4/14 (24%) used both amiodarone and sotalol (with greater proportional use of amiodarone compared to sotalol) and 3/14 (21%) used sotalol only. Studies’ overall RoB was evaluated as “Low” in 8/14 (57%) of trials, “Some concerns” in 4/14 (29%) and “High” in 2/14 (14%) of studies (Fig. 1; Table S.13).

**Table 1:** Study characteristics of included records

First author, Year	Follow-up (months)	Population	Prevention type	Intervention	Control	Total (n)	Intervention (*n*)	Control(n)
Al-Khatib (2015) CALYPSO[27]	6	Adults (≥ 18 years) + ICD + CAD + documented VT	Secondary	CA	Amiodarone/Sotalol	25	13	12
Arenal (2022) SURVIVE-VT [19]	24	Adults (≥ 18 years) + ICD + CAD (MI) + documented VT	Secondary	CA	Amiodarone 86%/Sotalol 14%	144	71	73
Della Bella (2023) PARTITA [28]	28,8	Adults (≥ 18 years) + ICD + documented VT	Secondary	CA	SC	45	23	24
Kuck (2010) VTACH [29]	22,5 (9)	Adults (18–80 years) + ICD + CAD (MI with LVEF ≤ 50%) + documented stable VT	Secondary	CA	SC	107	52	55
Kuck (2017) SMS [30]	27,6 (13,2)	Adults (18–80 years) + ICD + CAD (LVEF ≤ 40%) + documented unstable VT	Secondary	CA	SC	111	54	57
Reddy (2007) SMASH-VT [31]	22,5 (5,5)	Adults (≥ 18 years) + ICD + CAD (MI) + documented VT	Secondary	CA	SC	128	64	64
Sapp (2016) VANISH [32]	27,9 (17,1)	Adults (≥ 18 years) + ICD + CAD (MI) + documented VT	Secondary	CA	Amiodarone ± Mexiletine	259	132	127
Žižek d (2024) PREVENTIVE [33]	44,7 (20,7)	Adults (≥ 18 years) + ICD + CAD (MI)	Primary	CA	SC	60	30	30
Sapp (2024) VANISH 2 [11]	51,6	Adults (≥ 18 years) + ICD + CAD (MI) + documented VT	Secondary	CA	Amiodarone 51%/Sotalol 49%	416	203	213
Seidl (1998) [34]	26 (16)	Adults (≥ 18 years) + ICD + documented VT/VF	Secondary	Sotalol	SC	70	35	35
Pacifico (1999) Sotalol ICD Study [35]	12	Adults (≥ 18 years) + ICD + documented VT	Secondary	Sotalol	SC	302	151	151
Kettering (2002) [36]	24 (10,2)	Adults (18–80 years) + ICD + documented VT	Secondary	Sotalol	SC	100	50	50
Connolly (2006) OPTIC [9]	11,8 (3,2)	Adults (≥ 18 years) + ICD + LVEF ≤ 40% + documented VT/VF	Secondary	Amiodarone/ Sotalol	SC	412	274	138
Raatikainen (2025) MANTRA-VT [12]	24	Adults (≥ 18 years) + ICD + CAD (MI) + 2 documented VT/VF	Secondary	CA	Amiodarone 93%/Sotalol 7%	58	28	30

Abbreviations: *ICD* Implantable cardioverter defibrillator; *CAD* Coronary artery disease; *VT*, Ventricular tachycardia; *CA* Catheter ablation; *MI* Myocardial infarction; *SC* Standard care; *AAD* Antiarrhythmic drugs; *LVEF* Left ventricular ejection fraction; *VF* Ventricular fibrillation;

### Outcomes

**Fig. 2:**
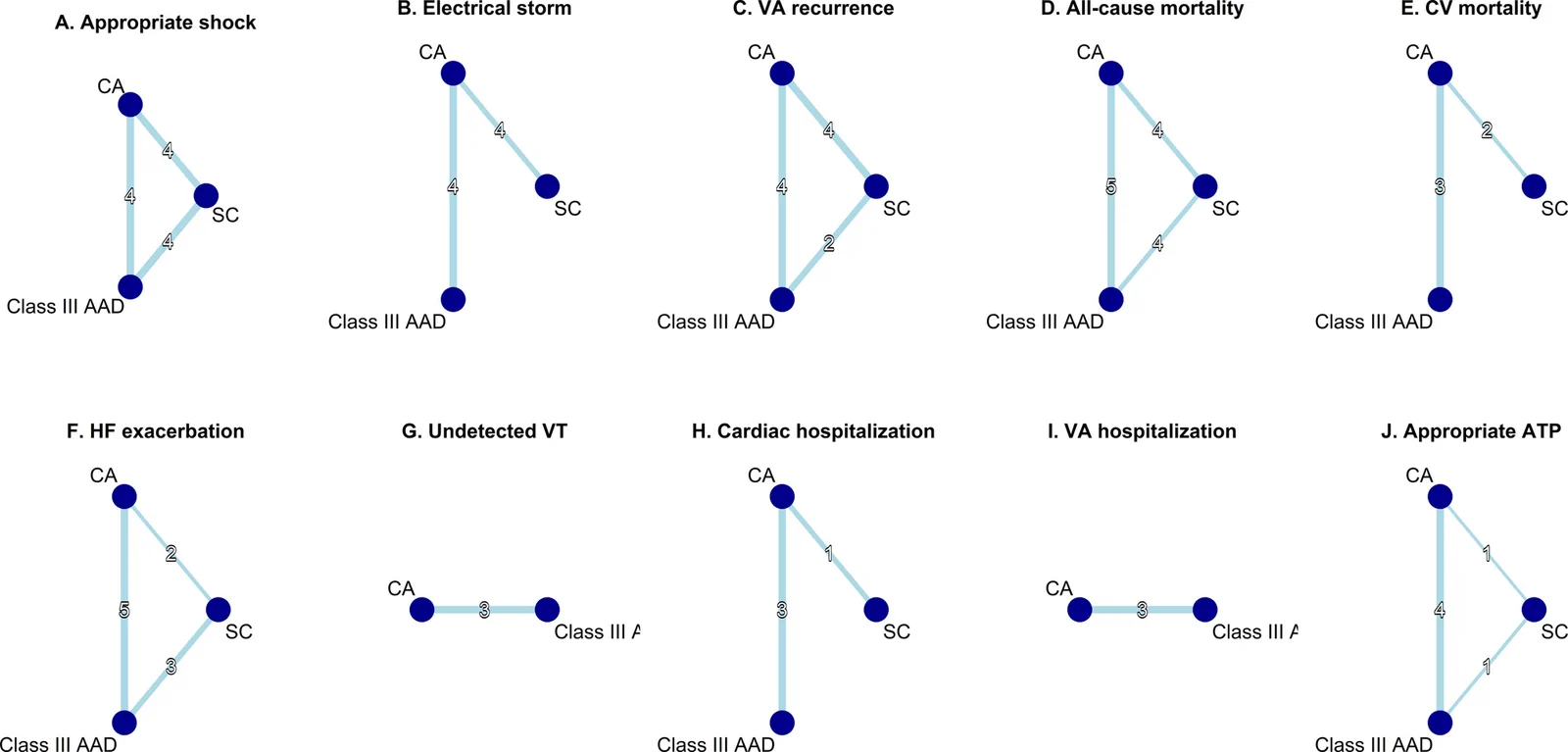
Network graphs for efficacy outcomes. Numbers on lines represent the number of studies evaluating a certain comparison. Linewidth corresponds to the standard error of the effect estimate of the direct pairwise comparison. Abbreviations: RR, risk ratio; CI, confidence interval; CA, catheter ablation; AAD, antiarrhythmic drugs; SC, standard care; VA, ventricular arrhythmia; CV, cardiovascular; HF, heart failure; VT, ventricular tachycardia; ATP, antitachycardia pacing

**Fig. 3:**
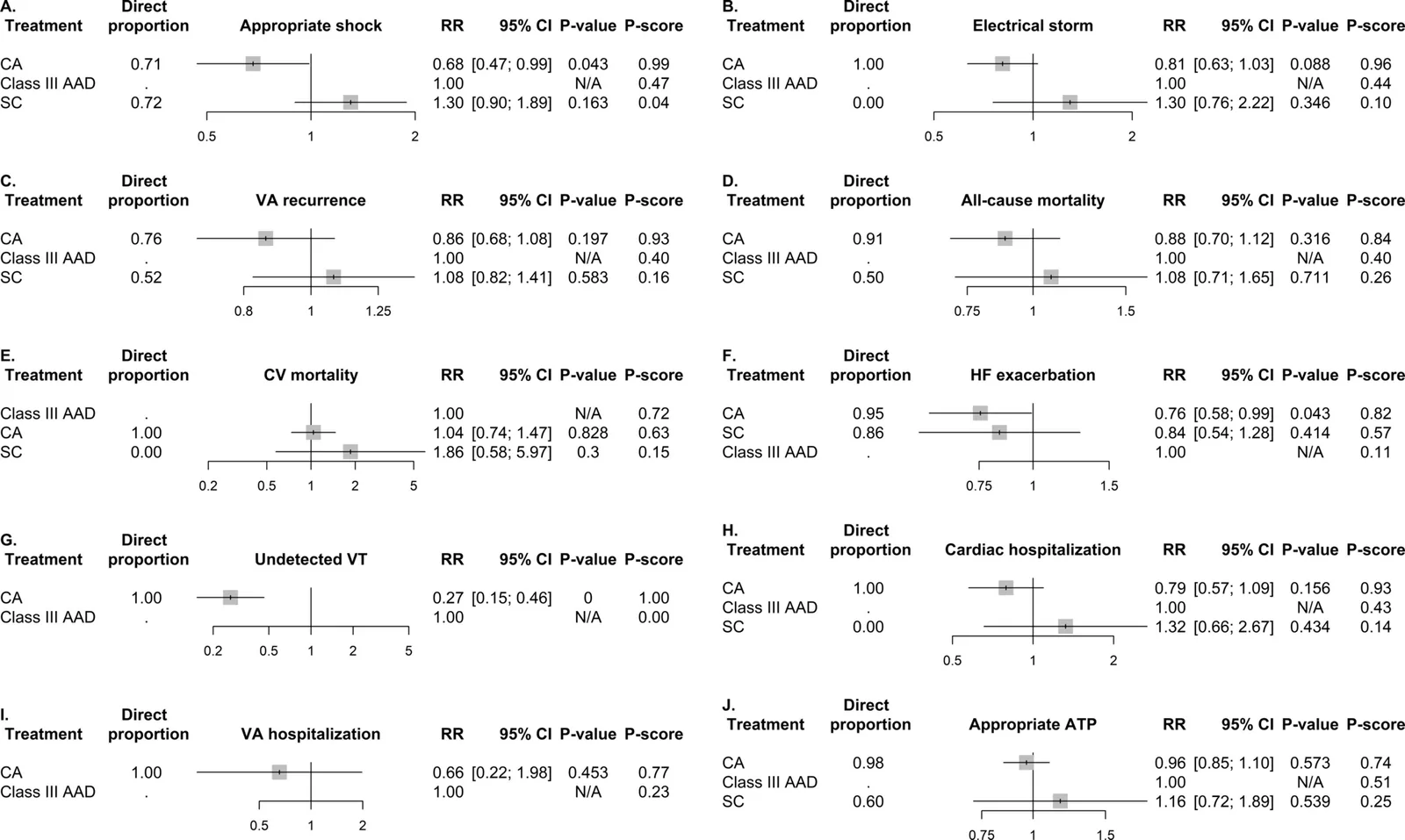
Forest plots for efficacy outcomes in the main analysis. All comparisons are against class III AAD as reference. Abbreviations: RR, risk ratio; CI, confidence interval; CA, catheter ablation; AAD, antiarrhythmic drugs; SC, standard care; VA, ventricular arrhythmia; CV, cardiovascular; HF, heart failure; VT, ventricular tachycardia; ATP, antitachycardia pacing

**Fig. 4:**
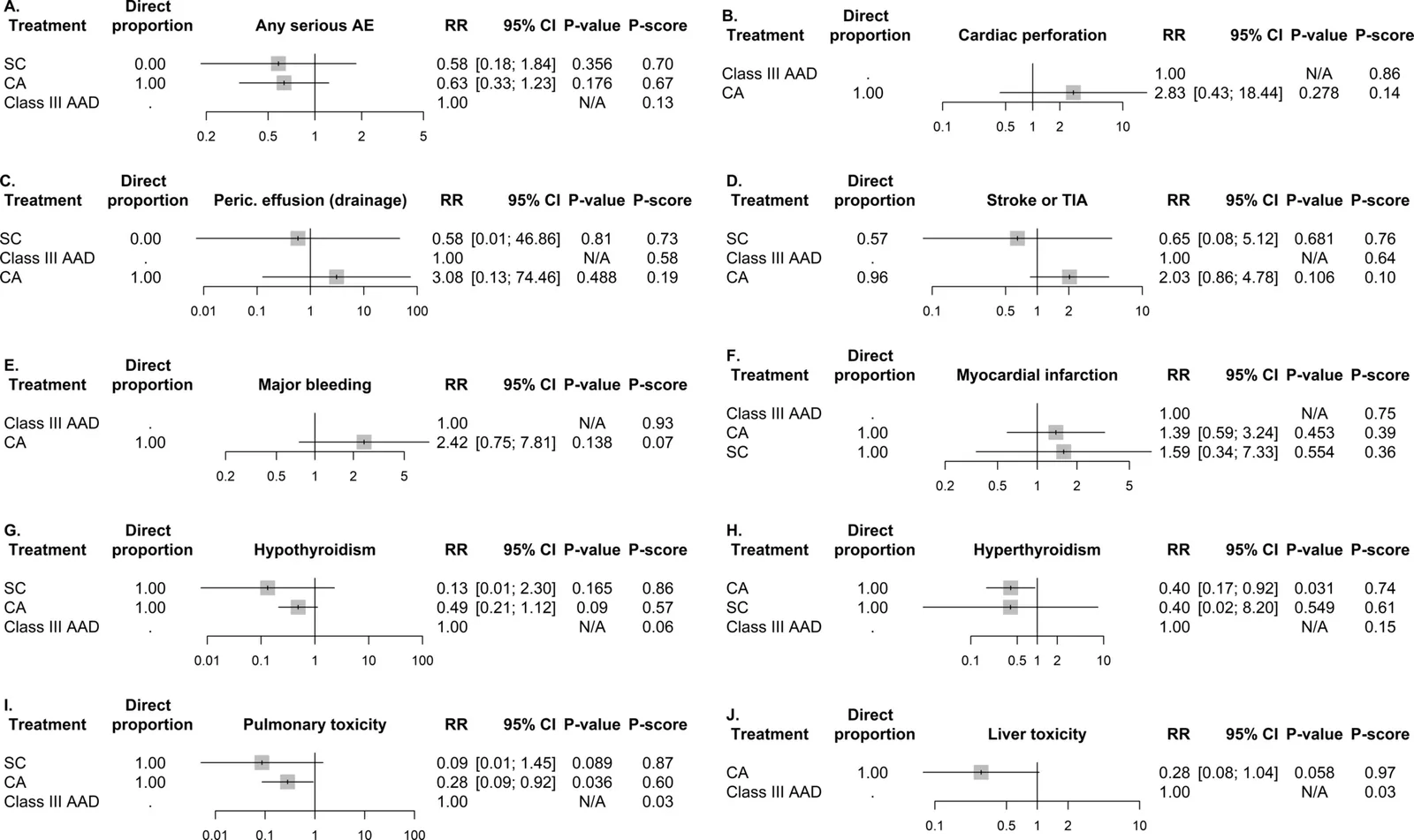
Forest plots for safety outcomes in the main analysis. All comparisons are against class III AAD as reference. Abbreviations: RR, risk ratio; CI, confidence interval; CA, catheter ablation; AAD, antiarrhythmic drugs; SC, standard care; AE, adverse event; TIA, transient ischemic attack; Peric., pericardial

 Network plots are presented in Fig. 2 for efficacy outcomes, and in Figure S.2 for safety outcomes. The forest plots of efficacy outcomes are presented in Fig. 3, and the corresponding plots for safety outcomes are found in Fig. 4. For the primary analysis, network information for all outcomes is summarized in Table 2 and league tables (containing all the pairwise estimates between interventions for each outcome) in Table S.14. Comparison-adjusted funnel plots are presented in Figure S.3 for efficacy outcomes and Figure S.4 for safety outcomes. Contribution plots for all outcomes (presenting the relative contribution of the direct and indirect estimates on the overall network estimate) are presented in Figures S.5-S.24. The characteristics of the outcome networks for the secondary analysis (splitting AAD into amiodarone and sotalol) are presented in Table S.15 and the corresponding league tables in Table S.16.

**Table 2:** Network parameters for each outcome

Outcome	Studies (*n*)	Participants (*n*)	Events (*n*)	Interventions	I^2^ (95% CI)	p_heterogeneity_	p_inconsistency_
A. Efficacy outcomes							
Appropriate shock	12	2154	577	3	0.56 (0.14, 0.78)	0.011	0.249
Electrical storm	8	1270	257	3	0.26 (0, 0.68)	0.229	N/A
VA recurrence	10	1208	575	3	0.49 (0, 0.76)	0.065	0.119
All-cause mortality	13	2181	290	3	0 (0, 0.58)	0.506	0.257
CV mortality	5	977	123	3	0 (0, 0.85)	0.637	N/A
HF exacerbation	10	1893	254	3	0 (0, 0.65)	0.831	0.136
Undetected VT	3	819	73	2	0 (0, 0.9)	N/A	N/A
Cardiac hospitalization	4	926	376	3	0.57 (0, 0.88)	0.099	N/A
VA hospitalization	3	229	43	2	0.66 (0, 0.9)	N/A	N/A
Appropriate ATP	6	1024	460	3	0.29 (0, 0.72)	0.169	0.454
B. Safety outcomes							
Any serious AE	4	698	225	3	0.63 (0, 0.89)	0.068	N/A
Cardiac perforation	2	675	5	2	0	N/A	N/A
Pericardial effusion (drainage)	2	255	3	3	N/A	N/A	N/A
Stroke/TIA	5	1030	25	3	0 (0, 0.85)	0.802	0.638
Major bleeding	2	675	14	2	0	N/A	N/A
Myocardial infarction	6	1516	28	3	0 (0, 0.79)	0.675	N/A
Hypothyroidism	5	1289	30	3	0 (0, 0.85)	0.965	N/A
Hyperthyroidism	4	1231	28	3	0 (0, 0.9)	0.813	N/A
Pulmonary toxicity	5	1289	25	3	0 (0, 0.85)	0.919	N/A
Liver toxicity	2	675	17	2	0.07	N/A	N/A

Abbreviations: *N/A* Not applicable; *CI* Confidence interval; *VA* Ventricular arrhythmia; *CV* Cardiovascular; *HF* Heart failure; *VT* Ventricular tachycardia; *ATP* Antitachycardia pacing; *AE* Adverse event; *TIA* Transient ischemic attack

#### Efficacy outcomes

Regarding appropriate shock (12 studies, 2154 participants; I^2^ = 56%; p_inconsistency_ = 0.249; Table 2; Fig. 3A), CA ranked first with significant differences against AAD (RR = 0.68, 95%CI = [0.47, 0.99]; *p* = 0.043; P-score = 0.99), mainly driven by the comparison versus sotalol (RR = 0.42, 95%CI = [0.26,0.69]; Table S.16), with no difference versus amiodarone (RR = 0.91, 95%CI = [0.57,1.46]; Table S.16). In ES (8 studies; 1270 participants; I^2^ = 26%; Table 2; Fig. 3B), CA ranked first but was not superior against AAD (RR = 0.81, 95%CI = [0.63,1.03]; *p* = 0.088; P-score = 0.96). No evidence of small study effect was identified for any primary outcome (funnel plots in Figure S.3).

In VA recurrence (10 studies, 1208 participants; I^2^ = 49%; p_inconsistency_ = 0.119; Table 2; Fig. 3C), no significant differences were found between CA and AAD (RR = 0.86, 95%CI = [0.68,1.08]; *p* = 0.197, P-score = 0.93). Regarding all-cause mortality, (13 studies, 2181 participants; I^2^ = 0%; p_inconsistency_ = 0.257; Table 2; Fig. 3D), CA did not differ significantly from AAD (RR = 0.88, 95%CI = [0.70,1.12]; p = 0.316, P-score = 0.84). In CV mortality (5 studies, 977 participants; I^2^ = 0%; Table 2; Fig. 3E), CA was also not superior to AAD (RR = 1.04, 95%CI = [0.74,1.47]; *p* = 0.828, P-score = 0.63). Regarding HF exacerbation (10 studies, 1893 participants; I^2^ = 0%; p_inconsistency_ = 0.136; Table 2; Fig. 3F), CA was superior to AAD (RR = 0.76, 95%CI = [0.58,0.99]; *p* = 0.043, P-score = 0.82), mainly due to the comparison versus amiodarone (RR = 0.67, 95%CI = [0.41,1.11]; Table S.16) and less so against sotalol (RR = 0.82, 95%CI = [0.43,1.55]; Table S.16).

Concerning the outcome of undetected VT-below ICD detection limit (3 studies, 819 participants; I^2^ = 0%; Table 2; Fig. 3G), CA ranked first and was superior to AAD (RR = 0.27, 95%CI = [0.15,0.46]; *p* < 0.001, P-score = 1) both against amiodarone (RR = 0.27, 95%CI = [0.15,0.49]; Table S.17) and sotalol (RR = 0.25, 95%CI = [0.13,0.49]; Table S.17). Of note, all three studies contributing to undetected VT (SURVIVE-VT, VANISH, VANISH-2) had programmed a VT-1 therapy zone ranging from 15–20 bpm lower than the slowest documented VT to a minimum of 130–150 bpm coupled with ATP therapies. Regarding hospitalization for cardiac causes (4 studies, 926 participants; I^2^ = 57%; Table 2; Fig. 3H), CA did not differ against AAD (RR = 0.79, 95%CI = [0.57,1.09]; p = 0.156; P-score = 0.93). In VA hospitalization (3 studies, 229 participants; I^2^ = 66%; Table 2; Fig. 3I), CA was not superior against AAD (RR = 0.66, 95%CI = [0.22,1.98]; *p* = 0.453, P-score = 0.77), while also no significant differences were found in appropriate ATP reduction (6 studies, 1024 participants; I^2^ = 29%; p_inconsistency_ = 0.454; Table 2) between these interventions (RR = 0.96, 95%CI = [0.85,1.10]; *p* = 0.573, P-score = 0.74).

#### Safety outcomes

Regarding the primary safety outcome of any serious AE (4 studies, 698 participants; I^2^ = 63%; Table 2; Fig. 4A) no difference was identified between CA and AAD (RR = 0.63, 95%CI = [0.33,1.23]; *p* = 0.176, P-score = 0.67) in the primary analysis. In the secondary analysis, CA was superior to amiodarone (RR = 0.33, 95%CI = [0.15,0.75]; Table S.16) and neutral against sotalol (RR = 0.39, 95%CI = [0.12,1.32]; Table S.16). For all the rest CA-related safety outcomes, an increased but nonsignificant risk was found for CA versus AAD in cardiac perforation, pericardial effusion requiring drainage, stroke/TIA, major bleeding and MI (Fig. 4). Regarding the AAD-related safety events, CA was associated with a smaller risk of hyperthyroidism (RR = 0.40, 95%CI = [0.17,0.92]; *p* = 0.031, P-score = 0.74) and pulmonary toxicity (RR = 0.28, 95%CI = [0.09,0.92]; *p* = 0.036, P-score = 0.60), driven by the comparison against amiodarone (Table S.16). Marginally nonsignificant differences were found in hypothyroidism and liver toxicity (Fig. 4G and J respectively).

### Sensitivity analyses

The results of the sensitivity analyses are shown in Figures S.25-S.34. In general, no noteworthy differences were observed regarding either the ranking of interventions or the magnitude and the direction effect estimates in all efficacy and safety outcomes. A table summarizing the exact studies that were analyzed in the main and sensitivity analyses is presented in Table S.17.

## Discussion

### Summary of findings in the context of other evidence

This is the first NMA to summarize the existing evidence by appropriately analyzing each individual intervention separately. Previous meta-analyses for VT ablation in ICM have provided effect estimates of CA versus control groups that comprised both AAD and SC into one group [10, 37, 38]. In contrast to this, using a NMA methodology, we split SC from AAD in our primary analysis, and we separately analyzed amiodarone and sotalol in our secondary analysis rendering our results clinically relevant and interpretable. In short, the findings of our main analysis suggest that in patients with ICM and ICD for secondary VA prevention, CA is superior to AAD in terms of efficacy with comparable safety. Indeed, CA was superior to AAD in reducing appropriate shocks, undetected VT and HF exacerbation. The effect on appropriate shocks was mainly versus sotalol, while in HF, versus amiodarone. The superiority in undetected VT was consistent against both amiodarone and sotalol.

Ablation was associated with a 19% reduction in ES and a 14% reduction in VA recurrence, though both effects were nonsignificant. It is possible, however, that patients presenting with ES might benefit more from CA as compared to patients with at least one documented VA (which was our inclusion criterion) based on the results of a recently published meta-analysis [39]. The neutral effect on total VA recurrences may be partly explained by the neutral effect on appropriate ATP reduction, which accounted for a larger proportion of appropriate ICD therapies compared to shocks (47% vs 33%) in our study. Interestingly, the reductions in appropriate shocks and undetected VT are directly associated with the benefits in HF exacerbation. Shocks, even when appropriate, do cause myocardial stunning, often leading to hospitalization and cardiac decompensation while worsening prognosis [40, 41]. Finally, undetected VT – often associated with extensive fibrosis and scar-related reentry circuits with multiple exits [42], as well as AAD treatment [43]– may turn into VF or lead to cardiac decompensation, itself a trigger for VAs, leading to a vicious cycle.

In our analysis, CA did not significantly impact all-cause or cardiovascular mortality. This finding aligns with previous evidence indicating that recurrent VA—whose incidence was not significantly reduced by ablation—remains a major determinant of adverse prognosis. In VANISH and VANISH-2 trials-which constitute the largest RCTs in the field-the significant effect on the primary composite endpoints were mainly driven by other co-primary endpoints and not from all-cause mortality. Similar results have been reported in the PAUSE-SCD trial that included patients with ischemic and non-ischemic cardiomyopathy. In this study ablation efficacy was driven by arrhythmia reduction rather than CV hospitalization or mortality [44]. This observation might be partly explained by reductions already achieved through optimal medical and interventional therapy [45]. The timing of CA could also serve as a significant effect modifier. Indeed, the recently published PREVENTIVE-VT trial [33] demonstrated considerable efficacy of CA in the primary prevention of arrhythmias, reducing not only arrhythmia-related, but also major clinical outcomes. Accordingly, in 528 patients treated with CA, age, NYHA class and ES were independent predictors of cardiac mortality after VT ablation [46], while based on the PAINESD score [47], age, NYHA class and LVEF constitute predictors of hemodynamic decompensation. Collectively, these data mandate for early intervention.

Regarding AEs, it is noteworthy that VT ablation (despite its complexity and application in morbid patients) demonstrated comparable safety against AAD in several treatment-related and serious AE, including stroke, need for pericardiocentesis, vascular complications and major bleeding. Interestingly, it was also superior against amiodarone regarding serious AE, while amiodarone was also associated with a higher risk of hyperthyroidism and pulmonary toxicity. Despite an overall rate of complications exceeding 13% [48, 49], temporal trends in major complications appear to remain stable ranging between 4%-8% [48, 50, 51]. Most frequent complications are associated with vascular access site (58% of major complications) [50], while other complications (including cardiac tamponade) appear to rise sharply when epicardial procedures are also included [48]. However, recent advancements have significantly improved the safety of epicardial access for VT ablation. Among them, the carbon dioxide (CO₂) insufflation technique, as described by Santangeli et al. [52], represents a major procedural innovation. In this approach, intrapericardial CO2 insufflation creates a pericardial separation that facilitates needle entry, minimizing myocardial or coronary injury, and inadvertent entry into adjacent structures.

Factors associated with major complications include [48, 50, 51]: structural heart disease (especially ICM), ES, non-elective procedures, and biventricular access. Mortality in the early post-ablation period (in-hospital and 30-day) ranges from 5% in older cohorts to 1.8% in newer studies, is mostly associated with arrhythmia recurrence (44.5%-80%) [48, 51] and is further increased when bail-out mechanical circulatory support is implemented (up to 88% within 10 days) [53]. Conversely, prophylactic circulatory support was associated with 33% reduced death/transplantation rates at 12 months post-ablation [54]. The Risk In Vt Ablation (RIVA) score may predict major complications and in-hospital mortality, with estimates ranging from < 5% to > 50% [48]. The positive association between operator’s experience and procedural safety should also be accounted in complex VT ablation procedures [55].

It is noteworthy that our results could have underestimated the effectiveness of CA. Class III AAD have been compared against SC in older trials where background therapy for HF and revascularization techniques were inferior, thus their additional effect on outcomes was more pronounced. Moreover, despite pharmacological stagnation over the 27-year span from the first included study (1998–2025), CA has shown significant advancements [56]. A paradigm shift has been observed from activation mapping to substrate modification with improved outcomes for patients with structural heart disease [57]. However, substrate ablation, when performed at all, was not based on high-density mapping and was limited to potential channel identification and pace-based mapping confirmation. A more modern elaboration of this approach is functional substrate mapping, where potential critical points for ablation are identified based on electrogram properties. Most variations are based on late potentials and local abnormal ventricular activities (LAVA) abolition (including potentials evoked during rapid pacing – decrement-evoked potentials – DeEP approach) and on ablating perpendicular to wavefront propagation in areas with isochronal confluence (isochronal latest activation mapping – ILAM) [58, 59]. Moreover, the introduction of high-density mapping and the notion of omnipolar potentials using grid catheters allows for better discrimination between near- and far-field signals and improved determination of wavefront propagation, facilitating the identification of conduction channels and VT exit sites [60]. To summarize, this technology dependence must be considered when evaluating the findings of our meta-analysis, where older studies may no longer reflect current practice outcomes due to the use of outdated technology.

### Implications for practice and research

The present NMA suggests that CA has superior efficacy with similar safety compared to AADs, which is particularly relevant in the era of prolonged survival of ICM patients with need for long-term VA suppression. The same applies in ES, justifying the ESC 2015 recommendations granting CA a first-line treatment status in dedicated centers [61]. Current guidelines recommend the use of catheter ablation in preference to escalating AAD therapy in patients with CAD and recurrent sustained VT despite amiodarone therapy. This recommendation stems mainly from the VANISH trial where a strategy of escalating antiarrhythmic medications was inferior to CA. Nevertheless, CA is not prioritized in patients solely on beta-blocker compared to AAD. Based on our results, the use of first-line CA over AAD in these patients seems reasonable, more so if there is a history or strong suspicion for VT below ICD detection threshold. Moreover, the reductions in HF exacerbation associated with CA merit particular attention. Even though HF was not a targeted outcome for most of the included studies, the robustness of our analysis and the lack of heterogeneity or inconsistency in this outcome increase the strength of our conclusions. Hence, the effect of CA on HF merits further consideration in future studies.

Recently published studies have highlighted the time-dependent efficacy of CA. A meta-analysis of seven RCT suggested that early CA was more beneficial compared to a deferred strategy [62]. In an even more revolutionary approach, the PREVENTIVE-VT trial demonstrated a significant benefit of CA for primary prevention in patients with ICM and chronic total coronary occlusions [33]. Hence, future research should focus on the effectiveness and safety of prophylactic CA in the modern era. Accordingly, the awaited results of the CASTLE-VT trial [63], where prophylactic CA in patients referred for heart transplantation will be tested, as well as those of the PREVENT-VT (NCT06543173) will shed more light on the field.

Further technological advancements may also improve outcomes for patients at risk for VA. A radical approach to CA lies in assessing the arrhythmogenic substrate using cardiac magnetic resonance [64, 65] or computed tomography [66] and even perform noninvasive programmed ventricular stimulation, to determine critical isthmuses pre-procedurally and minimize lesion formation [58, 67]. Additionally, the advent of digital twins could further facilitate the identification of the active part of a VT circuit [68]. Positive experience is also increasing with the implementation of artificial intelligence (AI) algorithms during the mapping procedure, suggesting the optimal ablation site [69, 70]. Finally, the use of pulsed-field ablation (PFA) in the field of VA [71–74] is emerging, given its ability to target cardiomyocytes specifically and the potential for more clearly delineated, deep lesion formation. In this direction, the promising results of VT ablation using a lattice-tip PFA catheter provided the first evidence of feasibility and safety in the AVAAR registry [75].

### Strengths and limitations

In general, we have conducted a rigorous analysis adhering to pertinent methodological and reporting guidelines and to a pre-registered protocol. The analysis of AAD as one group coupled with the separate analysis for amiodarone and sotalol further reinforce the clinical applicability of our results. Moreover, heterogeneity was minimal and the transitivity assumption seemed plausible for all outcomes, since statistical inconsistency (when feasible to be evaluated) was nonsignificant. Furthermore, no differences were found in the qualitative examination of the transitivity assumption via the distribution of potential effect modifiers across interventions and comparisons. Additionally, our results remained relatively robust in sensitivity analyses, whereas only a minority of the analyzed studies were at “High” RoB. Collectively, these observations increase the validity of our study.

Nonetheless, some limitations should also be acknowledged. In certain outcomes we followed the per-study definitions, which may impact the generalizability of our results. Technological advancements in contemporary CA practice might have also been underrepresented in our study, possibly leading to underestimation of CA efficacy. Additionally, variations were encountered in the reporting of VA events across studies and ICD programming was not uniform. Moreover, our analysis only included RCTs, which, despite being the gold standard for causal inference, usually fail to adequately represent the wide spectrum of different scenarios encountered in clinical practice. Additionally, despite the pooling of all available data from RCTs, our analysis might have been underpowered to detect significant effects in outcomes that were underrepresented by primary studies. Finally, additional analyses focusing on certain subgroups (e.g. patients with very low LVEF or patients presenting with ES) were not feasible due to lack of separate outcome reporting in individual studies.

## Conclusions

In conclusion, based on our main analysis, CA is superior in terms of efficacy with similar safety compared to class III AAD in reducing appropriate shocks, VT below ICD detection limit and HF exacerbation in patients with ICM and an ICD for secondary VA prevention. No significant reductions were observed in VA or cardiac hospitalization and all-cause or CV mortality. Notably, in our secondary analyses CA demonstrated superior efficacy over sotalol and superior safety over amiodarone. Given the plausibility of the transitivity assumption and the robustness of our results in sensitivity analyses, the conclusions of our study seem valid. New RCTs incorporating novel advances in the field of CA, including mapping (functional substrate, omnipolar potentials, virtual mapping and use of AI) and energy type (PFA) are necessary to determine the true potential and advantages of VT ablation over pharmacotherapy.

## Supplementary Information

Below is the link to the electronic supplementary material.

Supplementary file1 (DOCX 14582 KB)

## Data Availability

All data extracted and analyzed in this study can be made available upon reasonable request to the corresponding author.
