# Supplementary material for: Efficacy and safety of ventricular tachycardia ablation versus antiarrhythmics in ischemic cardiomyopathy: a network meta-analysis
Source: J Interv Card Electrophysiol. 2026 Feb 20;69(7):1871–84. doi: 10.1007/s10840-026-02250-9 (PMC13687245; doi:10.1007/s10840-026-02250-9)
Supplement: Supplementary file 1 [file 10840_2026_2250_MOESM1_ESM.docx]

Supplemental material

of

**Efficacy and Safety of Ventricular Tachycardia Ablation Versus Antiarrhythmics in Ischemic Cardiomyopathy: A Network Meta-Analysis**

Table of contents

**Supplemental Tables**

[**Table S.1.** Protocol amendments with rationale	4](#_Toc212968436)

[**Table S.2.** Checklist of reported items based on the PRISMA-NMA statement	5](#_Toc212968437)

[**Table S.3.** Full search strategy	9](#_Toc212968438)

[**Table S.4.** Outcome definitions across studies	11](#_Toc212968439)

[**Table S.5.** ICD programming parameters across studies	12](#_Toc212968440)

[**Table S.7.** Extracted variables	14](#_Toc212968441)

[**Table S.8.** Excluded studies with rationale	14](#_Toc212968442)

[**Table S.9.** Population characteristics of included records	15](#_Toc212968443)

[**Table S.10.** Mapping strategy and procedural endpoints in each VT ablation randomized clinical trial.	18](#_Toc212968444)

[**Table S.11.** Distribution of effect modifiers across different interventions	19](#_Toc212968445)

[**Table S.12.** Distribution of effect modifiers across different comparisons	21](#_Toc212968446)

[**Table S.13**. Detailed methodological quality assessments of included studies based on the Cochrane Risk of Bias v.2 tool	22](#_Toc212968447)

[**Table S.14**. League tables for main analysis	23](#_Toc212968448)

[**Table S.15**. Characteristics of outcome networks in the secondary analysis (splitting Class III AAD)	25](#_Toc212968449)

[**Table S.16**. League tables for secondary analysis (splitting Class III AAD)	25](#_Toc212968450)

[**Table S.17**. Studies included in each analysis	28](#_Toc212968451)

**Supplemental Figures**

[**Figure S.1.** Preferred Reporting Items for Systematic Reviews and Meta-Analyses (PRISMA) 2020 flowchart	30](#_Toc212968452)

[**Figure S.2.** Network graphs for safety outcomes	31](#_Toc212968453)

[**Figure S.3.** Comparison-adjusted funnel plots for efficacy outcomes accompanied by Egger’s test p-value.	32](#_Toc212968454)

[**Figure S.4.** Comparison-adjusted funnel plots for safety outcomes accompanied by Egger’s test p-value.	33](#_Toc212968455)

[**Figure S.5.** Contribution plot for the outcome of appropriate shock.	34](#_Toc212968456)

[**Figure S.6.** Contribution plot for the outcome of electrical storm.	34](#_Toc212968458)

[**Figure S.7.** Contribution plot for the outcome of VA recurrence.	35](#_Toc212968460)

[**Figure S.8.** Contribution plot for the outcome of all-cause mortality.	35](#_Toc212968462)

[**Figure S.9.** Contribution plot for the outcome of CV mortality.	36](#_Toc212968463)

[**Figure S.10.** Contribution plot for the outcome of HF exacerbation.	36](#_Toc212968464)

[**Figure S.11.** Contribution plot for the outcome of undetected VT.	37](#_Toc212968465)

[**Figure S.12.** Contribution plot for the outcome of cardiac hospitalization.	37](#_Toc212968466)

[**Figure S.13.** Contribution plot for the outcome of VA hospitalization.	37](#_Toc212968467)

[**Figure S.14.** Contribution plot for the outcome of appropriate ATP.	38](#_Toc212968468)

[**Figure S.15.** Contribution plot for the outcome of any serious AE.	38](#_Toc212968469)

[**Figure S.16.** Contribution plot for the outcome of cardiac perforation.	39](#_Toc212968470)

[**Figure S.17.** Contribution plot for the outcome of pericardial effusion (drainage).	39](#_Toc212968471)

[**Figure S.18.** Contribution plot for the outcome of stroke/TIA.	40](#_Toc212968472)

[**Figure S.19.** Contribution plot for the outcome of major bleeding.	40](#_Toc212968473)

[**Figure S.20.** Contribution plot for the outcome of myocardial infarction.	41](#_Toc212968474)

[**Figure S.21.** Contribution plot for the outcome of hypothyroidism.	41](#_Toc212968475)

[**Figure S.22.** Contribution plot for the outcome of hyperhtyroidism.	42](#_Toc212968476)

[**Figure S.23.** Contribution plot for the outcome of pulmonary toxicity.	42](#_Toc212968477)

[**Figure S.24.** Contribution plot for the outcome of liver toxicity.	43](#_Toc212968478)

[**Figure S.25.** Sensitivity analysis including both primary and secondary prevention trials (efficacy outcomes).	43](#_Toc212968479)

[**Figure S.26.** Sensitivity analysis including both primary and secondary prevention trials (safety outcomes).	44](#_Toc212968480)

[**Figure S.27.** Sensitivity analysis including only CAD patients (efficacy outcomes).	45](#_Toc212968481)

[**Figure S.28.** Sensitivity analysis including only CAD patients (safety outcomes).	46](#_Toc212968482)

[**Figure S.29.** Sensitivity analysis including only studies with n≥100 (efficacy outcomes).	47](#_Toc212968483)

[**Figure S.30.** Sensitivity analysis including only studies with n≥100 (safety outcomes).	48](#_Toc212968484)

[**Figure S.31.** Sensitivity analysis including studies that enrolled after 2000 (efficacy outcomes).	49](#_Toc212968485)

[**Figure S.32.** Sensitivity analysis including studies that enrolled after 2000 (safety outcomes).	50](#_Toc212968486)

[**Figure S.33.** Sensitivity analysis including studies that enrolled after MADIT-RIT (efficacy outcomes).	51](#_Toc212968487)

[**Figure S.34.** Sensitivity analysis including studies that enrolled after MADIT-RIT (safety outcomes).	52](#_Toc212968488)

[**References of the supplemental material**	53](#_Toc212968489)

## **Table S.1.** Protocol amendments with rationale

| **Amendment** | **Rationale** |
| --- | --- |
| Appropriate ATP therapy was included as an additional outcome | To more comprehensively assess the efficacy of interventions in arrhythmia recurrence and to elucidate the lack of effect of CA in total VA recurrence. |
| The outcome of any AE was not included | Data regarding any AE were inconsistently reported across studies. Some studies reported only individual AE without an overall pool or solely treatment-related AE. Hence a valid analysis for this outcome was not feasible. |
| Several safety outcomes were included | Due to inconsistent reporting of data for any AE and to further investigate the safety of interventions, several additional safety outcomes were included. |
| In the primary outcomes, VA recurrence was substituted with appropriate shock. | Data on VA recurrence were not uniformly reported across studies. Some studies reported only appropriate ICD therapies, while others reported both appropriate ICD therapies and undetected sustained (≥30s) VA (below detection limit). Moreover, the effect on overall appropriate shocks could be more clinically relevant for the population examined as compared to overall VA (which includes the painless termination by ATP). Appropriate shock was also more consistently reported across studies. |
| An additional secondary analysis was performed by splitting Class III AAD into amiodarone and sotalol. | A recently published report of the VANISH-2 trial allowed the extraction of events separately for amiodarone and sotalol. This analysis provided deeper insights into the comparative effectiveness and safety of interventions in secondary prevention patients. |
| Additional sensitivity analyses were performed based on the timeline of the implementation of HF treatment and the publication of the MADIT-RIT trial that influenced ICD programming | These analyses provide further insights on the robustness of our conclusions. |

**Abbreviations:** ATP, antitachycardia pacing; AAD, antiarrhythmic drugs; AE, adverse events; ICD, implantable cardioverter defibrillator; CA, catheter ablation; VA, ventricular arrhythmia;

## **Table S.2.** Checklist of reported items based on the PRISMA-NMA statement

| **Section/Topic** | **Item #** | **Checklist Item** | **Reported on Page #** |
| --- | --- | --- | --- |
| **TITLE** |  |  |  |
| Title | 1 | Identify the report as a systematic review *incorporating a network meta-analysis (or related form of meta-analysis).* | 1 |
|  |  |  |  |
| **ABSTRACT** |  |  |  |
| Structured summary | 2 | Provide a structured summary including, as applicable:  **Background:** main objectives  **Methods:** data sources; study eligibility criteria, participants, and interventions; study appraisal; and *synthesis methods, such as network meta-analysis.*  **Results:** number of studies and participants identified; summary estimates with corresponding confidence/credible intervals; *treatment rankings may also be discussed. Authors may choose to summarize pairwise comparisons against a chosen treatment included in their analyses for brevity.*  **Discussion/Conclusions:** limitations; conclusions and implications of findings.  **Other:** primary source of funding; systematic review registration number with registry name. | 3-4 |
|  |  |  |  |
| **INTRODUCTION** |  |  |  |
| Rationale | 3 | Describe the rationale for the review in the context of what is already known*, including mention of why a network meta-analysis has been conducted.* | 6-7 |
| Objectives | 4 | Provide an explicit statement of questions being addressed, with reference to participants, interventions, comparisons, outcomes, and study design (PICOS). | 7 |
|  |  |  |  |
| **METHODS** |  |  |  |
| Protocol and registration | 5 | Indicate whether a review protocol exists and if and where it can be accessed (e.g., Web address); and, if available, provide registration information, including registration number. | 7 |
| Eligibility criteria | 6 | Specify study characteristics (e.g., PICOS, length of follow-up) and report characteristics (e.g., years considered, language, publication status) used as criteria for eligibility, giving rationale. *Clearly describe eligible treatments included in the treatment network, and note whether any have been clustered or merged into the same node (with justification).* | 7-8 |
| Information sources | 7 | Describe all information sources (e.g., databases with dates of coverage, contact with study authors to identify additional studies) in the search and date last searched. | 7 |
| Search | 8 | Present full electronic search strategy for at least one database, including any limits used, such that it could be repeated. | Table S.3 |
| Study selection | 9 | State the process for selecting studies (i.e., screening, eligibility, included in systematic review, and, if applicable, included in the meta-analysis). | 7-8 |
| Data collection process | 10 | Describe method of data extraction from reports (e.g., piloted forms, independently, in duplicate) and any processes for obtaining and confirming data from investigators. | 8-9 |
| Data items | 11 | List and define all variables for which data were sought (e.g., PICOS, funding sources) and any assumptions and simplifications made. | 9 |
| **Geometry of the network** | **S1** | Describe methods used to explore the geometry of the treatment network under study and potential biases related to it. This should include how the evidence base has been graphically summarized for presentation, and what characteristics were compiled and used to describe the evidence base to readers. | 9-10 |
| Risk of bias within individual studies | 12 | Describe methods used for assessing risk of bias of individual studies (including specification of whether this was done at the study or outcome level), and how this information is to be used in any data synthesis. | 10  Table S.13 |
| Summary measures | 13 | State the principal summary measures (e.g., risk ratio, difference in means). *Also describe the use of additional summary measures assessed, such as treatment rankings and surface under the cumulative ranking curve (SUCRA) values, as well as modified approaches used to present summary findings from meta-analyses.* | 10 |
| Planned methods of analysis | 14 | Describe the methods of handling data and combining results of studies for each network meta-analysis. This should include, but not be limited to:   - *Handling of multi-arm trials;* - *Selection of variance structure;* - *Selection of prior distributions in Bayesian analyses; and* - *Assessment of model fit.* | 10-11 |
| **Assessment of Inconsistency** | **S2** | Describe the statistical methods used to evaluate the agreement of direct and indirect evidence in the treatment network(s) studied. Describe efforts taken to address its presence when found. | 10-11 |
| Risk of bias across studies | 15 | Specify any assessment of risk of bias that may affect the cumulative evidence (e.g., publication bias, selective reporting within studies). | 11 |
| Additional analyses | 16 | Describe methods of additional analyses if done, indicating which were pre-specified. This may include, but not be limited to, the following:   - Sensitivity or subgroup analyses; - Meta-regression analyses; - *Alternative formulations of the treatment network; and* - *Use of alternative prior distributions for Bayesian analyses (if applicable).* | 9, 11 |
| **RESULTS†** |  |  |  |
| Study selection | 17 | Give numbers of studies screened, assessed for eligibility, and included in the review, with reasons for exclusions at each stage, ideally with a flow diagram. | 11  Table S.8 |
| **Presentation of network structure** | **S3** | Provide a network graph of the included studies to enable visualization of the geometry of the treatment network. | 12 |
| **Summary of network geometry** | **S4** | Provide a brief overview of characteristics of the treatment network. This may include commentary on the abundance of trials and randomized patients for the different interventions and pairwise comparisons in the network, gaps of evidence in the treatment network, and potential biases reflected by the network structure. | 12 |
| Study characteristics | 18 | For each study, present characteristics for which data were extracted (e.g., study size, PICOS, follow-up period) and provide the citations. | 11-12  Table 1 |
| Risk of bias within studies | 19 | Present data on risk of bias of each study and, if available, any outcome level assessment. | 12  Table S.13 |
| Results of individual studies | 20 | For all outcomes considered (benefits or harms), present, for each study: 1) simple summary data for each intervention group, and 2) effect estimates and confidence intervals. *Modified approaches may be needed to deal with information from larger networks.* | 12-14 |
| Synthesis of results | 21 | Present results of each meta-analysis done, including confidence/credible intervals. *In larger networks, authors may focus on comparisons versus a particular comparator (e.g. placebo or standard care), with full findings presented in an appendix. League tables and forest plots may be considered to summarize pairwise comparisons.* If additional summary measures were explored (such as treatment rankings), these should also be presented. | 12-14 |
| **Exploration for inconsistency** | **S5** | Describe results from investigations of inconsistency. This may include such information as measures of model fit to compare consistency and inconsistency models, *P* values from statistical tests, or summary of inconsistency estimates from different parts of the treatment network. | N/A |
| Risk of bias across studies | 22 | Present results of any assessment of risk of bias across studies for the evidence base being studied. | N/A |
| Results of additional analyses | 23 | Give results of additional analyses, if done (e.g., sensitivity or subgroup analyses, meta-regression analyses*, alternative network geometries studied, alternative choice of prior distributions for Bayesian analyses,* and so forth). | 14-15 |
|  |  |  |  |
| **DISCUSSION** |  |  |  |
| Summary of evidence | 24 | Summarize the main findings, including the strength of evidence for each main outcome; consider their relevance to key groups (e.g., healthcare providers, users, and policy-makers). | 15-20 |
| Limitations | 25 | Discuss limitations at study and outcome level (e.g., risk of bias), and at review level (e.g., incomplete retrieval of identified research, reporting bias). *Comment on the validity of the assumptions, such as transitivity and consistency. Comment on any concerns regarding network geometry (e.g., avoidance of certain comparisons).* | 20-21 |
| Conclusions | 26 | Provide a general interpretation of the results in the context of other evidence, and implications for future research. | 21 |
|  |  |  |  |
| **FUNDING** |  |  |  |
| Funding | 27 | Describe sources of funding for the systematic review and other support (e.g., supply of data); role of funders for the systematic review. This should also include information regarding whether funding has been received from manufacturers of treatments in the network and/or whether some of the authors are content experts with professional conflicts of interest that could affect use of treatments in the network. | 21 |

Abbreviations: PICOS = population, intervention, comparators, outcomes, study design; PRISMA NMA, Preferred Reporting Items for Systematic Reviews and Meta-analyses extension for Network Meta-Analysis

## **Table S.3.** Full search strategy

**MEDLINE (via Pubmed)**

("tachycardia, ventricular"[MeSH Terms] OR "tachycardia ventricular*"[All Fields] OR "tachycardias ventricular*"[All Fields] OR "ventricular tachycardia*"[All Fields] OR "ventricular tachyarrhythmia*"[All Fields] OR "tachyarrhythmia ventricular*"[All Fields] OR "ventricular tachyarrhythmia*"[All Fields] OR "ventricular arrhythmia*"[All Fields] OR "Ventricular Fibrillation"[MeSH Terms] OR "ventricular fibrillation*"[All Fields] OR "electrical storm*"[All Fields] OR "defibrillators, implantable"[MeSH Terms] OR "defibrillator implantable*"[All Fields] OR "ICD"[All Fields] OR "implantable defibrillator*"[All Fields] OR "implantable cardioverter defibrillator*"[All Fields] OR "cardioverter defibrillator implantable*"[All Fields] OR "cardioverter defibrillators implantable*"[All Fields] OR "defibrillator implantable cardioverter*"[All Fields] OR "defibrillators implantable cardioverter*"[All Fields] OR "implantable cardioverter defibrillator*"[All Fields] OR "cardioverter defibrillators implantabl*"[All Fields] OR "cardioverter defibrillator implantabl*"[All Fields] OR "implantable cardioverter defibrillator*"[All Fields] OR "implantable cardioverter defibrillator*"[All Fields] OR "implantable defibrillators*"[All Fields]) AND ("Catheter Ablation"[MeSH Terms] OR "catheter ablation*"[All Fields] OR "ablation catheter*"[All Fields] OR "electric ablation*"[All Fields] OR "transvenous ablation*"[All Fields] OR "catheter ablation electric*"[All Fields] OR "electric catheter ablation*"[All Fields] OR "catheter ablation electrical*"[All Fields] OR "electrical catheter ablation*"[All Fields] OR "catheter ablation percutaneous*"[All Fields] OR "percutaneous catheter ablation*"[All Fields] OR "catheter ablation radiofrequenc*"[All Fields] OR "radiofrequency catheter ablation*"[All Fields] OR "ablation radiofrequency catheter*"[All Fields] OR "Radiofrequency ablation*"[All Fields] OR "RF ablation*"[All Fields] OR "pulsed field ablation*"[All Fields] OR "pulsed field ablation*"[All Fields] OR "cryoablation*"[All Fields] OR "cryothermal ablation*"[All Fields] OR "Anti-Arrhythmia Agents"[Pharmacological Action] OR "antiarrhythmic*"[All Fields] OR "anti arrhythmic*"[All Fields] OR "Amiodarone"[MeSH Terms] OR "amiodarone*"[All Fields] OR "cordarone*"[All Fields] OR "kordaron*"[All Fields] OR "cordarex*"[All Fields] OR "trangorex*"[All Fields] OR "amiodarona*"[All Fields] OR "angoron*"[All Fields] OR "aratac*"[All Fields] OR "amiodarone*"[All Fields] OR "Sotalol"[MeSH Terms] OR "Sotalol"[All Fields]) AND ("randomized controlled trial"[Publication Type] OR "controlled clinical trial"[Publication Type] OR ("randomized"[Title/Abstract] OR "randomised"[Title/Abstract]) OR "placebo"[Title/Abstract] OR "drug therapy"[MeSH Subheading] OR "randomly"[Title/Abstract] OR "trial"[Title/Abstract] OR "groups"[Title/Abstract])) NOT ("animals"[MeSH Terms] NOT "humans"[MeSH Terms]))

**Scopus**

(TITLE-ABS-KEY("tachycardia ventricular*") OR TITLE-ABS-KEY("tachycardias ventricular*") OR TITLE-ABS-KEY("ventricular tachycardia*") OR TITLE-ABS-KEY("ventricular tachyarrhythmia*") OR TITLE-ABS-KEY("tachyarrhythmia ventricular*") OR TITLE-ABS-KEY("ventricular arrhythmia*") OR TITLE-ABS-KEY("ventricular fibrillation*") OR TITLE-ABS-KEY("electrical storm*") OR TITLE-ABS-KEY("defibrillator implantable*") OR TITLE-ABS-KEY("ICD") OR TITLE-ABS-KEY("implantable defibrillator*") OR TITLE-ABS-KEY("implantable cardioverter defibrillator*") OR TITLE-ABS-KEY("cardioverter defibrillator implantable*") OR TITLE-ABS-KEY("cardioverter defibrillators implantable*") OR TITLE-ABS-KEY("defibrillator implantable cardioverter*") OR TITLE-ABS-KEY("defibrillators implantable cardioverter*") OR TITLE-ABS-KEY("implantable defibrillators*") ) AND ( TITLE-ABS-KEY("catheter ablation*") OR TITLE-ABS-KEY("ablation catheter*") OR TITLE-ABS-KEY("electric ablation*") OR TITLE-ABS-KEY("transvenous ablation*") OR TITLE-ABS-KEY("catheter ablation electric*") OR TITLE-ABS-KEY("electric catheter ablation*") OR TITLE-ABS-KEY("catheter ablation electrical*") OR TITLE-ABS-KEY("electrical catheter ablation*") OR TITLE-ABS-KEY("catheter ablation percutaneous*") OR TITLE-ABS-KEY("percutaneous catheter ablation*") OR TITLE-ABS-KEY("catheter ablation radiofrequenc*") OR TITLE-ABS-KEY("radiofrequency catheter ablation*") OR TITLE-ABS-KEY("ablation radiofrequency catheter*") OR TITLE-ABS-KEY("radiofrequency ablation*") OR TITLE-ABS-KEY("RF ablation*") OR TITLE-ABS-KEY("pulsed field ablation*") OR TITLE-ABS-KEY("cryoablation*") OR TITLE-ABS-KEY("cryothermal ablation*") OR TITLE-ABS-KEY("antiarrhythmic*") OR TITLE-ABS-KEY("anti arrhythmic*") OR TITLE-ABS-KEY("amiodarone*") OR TITLE-ABS-KEY("cordarone*") OR TITLE-ABS-KEY("kordaron*") OR TITLE-ABS-KEY("cordarex*") OR TITLE-ABS-KEY("trangorex*") OR TITLE-ABS-KEY("amiodarona*") OR TITLE-ABS-KEY("angoron*") OR TITLE-ABS-KEY("aratac*") OR TITLE-ABS-KEY("sotalol") ) AND(TITLE-ABS-KEY("randomized controlled trial") OR TITLE-ABS-KEY("controlled clinical trial") OR TITLE-ABS-KEY("randomized") OR TITLE-ABS-KEY("randomised") OR TITLE-ABS-KEY("placebo") OR TITLE-ABS-KEY("drug therapy") OR TITLE-ABS-KEY("randomly") OR TITLE-ABS-KEY("trial") OR TITLE-ABS-KEY("groups") OR TITLE-ABS-KEY("RCT"))

**Cochrane Library**

ID	Search	Hits

#1	MeSH descriptor: [Tachycardia, Ventricular] explode all trees	1005

#2	MeSH descriptor: [Defibrillators, Implantable] explode all trees	1505

#3	MeSH descriptor: [Ventricular Fibrillation] explode all trees	700

#4	(ventricular tachyarrhythmia):ti,ab,kw (Word variations have been searched)	663

#5	(ventricular tachycardia):ti,ab,kw (Word variations have been searched)	3276

#6	(ventricular fibrillation):ti,ab,kw (Word variations have been searched)	4693

#7	(implantable cardioverter defibrillator):ti,ab,kw (Word variations have been searched)	2866

#8	MeSH descriptor: [Amiodarone] explode all trees	857

#9	(amiodarone):ti,ab,kw (Word variations have been searched)	1884

#10	MeSH descriptor: [Sotalol] explode all trees	357

#11	(sotalol):ti,ab,kw (Word variations have been searched)	622

#12	MeSH descriptor: [Anti-Arrhythmia Agents] explode all trees	2430

#13	(antiarrhythmic):ti,ab,kw (Word variations have been searched)	3435

#14	MeSH descriptor: [Catheter Ablation] explode all trees	2410

#15	(catheter ablation):ti,ab,kw (Word variations have been searched)	4785

#16	(#1 OR #2 OR #3 OR #4 OR #5 OR #6 OR #7) AND (#8 OR #9 OR #10 OR #11 OR #12 OR #13 OR #14 OR #15)	2161

## **Table S.4.** Outcome definitions across studies

| **First author (Study name), Year** | **VA recurrence** | **Adverse events** | **Serious adverse events** |
| --- | --- | --- | --- |
| Al-Khatib AM (2015) CALYPSO[36] | - Sustained VT ≥30s - VT resulting in appropriate ICD therapy | N/R | N/R |
| Arenal A (2022) SURVIVE-VT [37] | - Appropriate ICD therapy - Monomorphic VT with a formal indication for ICD | Significant non-fatal events without need for hospitalization | Fatal events or non-fatal cardiovascular events prompting hospital admission or prolonging hospitalization. |
| Della Bella P (2023) PARTITA [38] | - Sustained (≥30s) VT or VF - Appropriate ICD therapy | Did not include data on any AE | Did not include data on serious AE |
| Kuck KH (2010) VTACH [39] | - Sustained (≥30s) VT or VF - Appropriate ICD therapy | Did not include data on any AE | Did not include data on serious AE |
| Kuck KH (2017) SMS [30] | - Sustained (≥30s) VT or VF - Appropriate ICD therapy | Did not include data on any AE | N/R |
| Reddy VY (2007) SMASH-VT [40] | - Appropriate ICD therapy | Did not include data on any AE | Did not include data on serious AE |
| Sapp JL (2016) VANISH [41] | Did not include data on overall VA recurrence. The primary outcome included appropriate ICD.  This study did not contribute in VA recurrences. | Treatment-related adverse event: Cardiovascular hospitalizations >24h or events related to treatment | Any event resulting in cardiovascular hospitalization for >24 hrs, or related to study treatment which resulted in hospitalization, prolongation of hospitalization, congenital malformation, persistent or significant disability/incapacity or were life-threatening. |
| Žižek d (2024) PREVENTIVE VT [42] | - Sustained (≥30s) VT or VF - Appropriate ICD therapy | Did not include data on any AE | Events attributed to CA requiring intervention or prolonged hospitalization (periprocedural death or cardiac arrest, vascular complications, oesophageal fistula, stroke/TIA, systemic embolism, pericardial perforation, pericardial effusion or tamponade requiring percutaneous or surgical intervention, MI, permanent heart block |
| Sapp JL (2024) VANISH 2 [43] | - Sustained (≥30s) VT - Appropriate ICD therapy | Safety events were those not meeting the criteria for a serious adverse event or involving thyroid or liver dysfunction.  Treatment-related adverse events were defined as those occurring within 30 days after catheter ablation, or events from antiarrhythmic drug therapy that led to discontinuation or dose reduction and were judged by the adjudication committee as definitely or likely related to the therapy. | Fatal events and cardiovascular events requiring admission to hospital, or prolonging hospitalization |
| Seidl K (1998) [44] | - Appropriate ICD therapy | Did not include data on any AE | Did not include data on serious AE |
| Pacifico A (1999) Sotalol ICD Study [45] | Did not include data on overall VA recurrence. The primary outcome included appropriate ICD.  This study did not contribute in VA recurrences. | Did not include data on any AE | Did not include data on serious AE |
| Kettering K (2002) [46] | Did not include data on overall VA recurrence. The secondary outcome included appropriate ICD shocks  This study did not contribute in VA recurrences. | Dizziness, Fatigue, Anxiety, Dyspnea, Peripheral edema, Pulmonary edema, HF, Postural hypotension, Bradycardia, Heart block, Prolongation of QT interval, Torsade de pointes, Chest pain, MI, Coronary revascularization. | |
| Connolly SJ (2006) OPTIC [47] | - Appropriate ICD therapy | Deaths, cardiac events (arrhythmic death, myocardial infarction, heart failure, atrial fibrillation), pulmonary events, thyroid dysfunction, symptomatic bradycardia, torsades de pointes, skin reactions, device infections, and hospitalizations. | Did not specify data on serious AE |
| Raatikainen P (2025) MANTRA-VT [48] | - Sustained (≥30s) VT/VF - Appropriate ICD therapy | Any unfavorable experience (sign, symptom, illness, abnormal lab result, or other medical event) occurring in a participant during the study, regardless of its relation to treatment. | Any clinical event causing death, life-threatening complication, significant or lasting disability, hospitalization or its prolongation, or requiring intervention to prevent permanent impairment or damage. |
| Abbreviations: VA, ventricular arrhythmia; VT, ventricular tachycardia; VF, ventricular fibrillation; ICD, implantable cardioverter-defibrillator; AE, adverse event; HF, heart failure; MI, myocardial infarction; TIA, transient ischemic attack; N/R, not reported. | | | |

## **Table S.5.** ICD programming parameters across studies

| **First author (Study name), Year** | **VT-1 zone** | **VT-2 zone** | **VF zone** |
| --- | --- | --- | --- |
| Al-Khatib AM (2015) CALYPSO[36] | - 20 bpm lower than VT-2 zone - Monitor only | - 10 bpm lower than the slowest documented VT | - ≥188 bpm |
| Arenal A (2022) SURVIVE-VT [37] | - 15-20 bpm lower than the slower documented VT up to a maximum of 188 bpm (320 ms) - ATP (4x10 at 91% CL) 🡪 max shock | - 320-260 ms - ATP (1x6 at 84% or 1x8 at 88% CL) 🡪 max shock | - <260 ms with max shock and ATP during charge |
| Della Bella P (2023) PARTITA [38] | - 130 bpm - Monitor only | - 167 bpm (360 ms) - ATP (2x8 bursts at 85% CL) 🡪 ATP (1x8 Ramp at 85% CL) 🡪 8 shocks | - 200 bpm (300 ms) - ATP during charging (1x8 bursts at 85% CL) 🡪 max shock |
| Kuck KH (2010) VTACH [39] | No VT-1 zone programmed | - 60 ms above the slowest documented VT - ATP 🡪 shock   (St Jude only) | - 200-220 bpm - ATP 🡪 shock   (St Jude only) |
| Kuck KH (2017) SMS [30] | No VT-1 zone programmed | - 160-180 bpm (if VT>220 bpm) or 60 ms lower than slower VT (if VT<220 bpm) - ATP 🡪 shock   (Medtronic only) | - 200-220 bpm - Shock only   (Medtronic only) |
| Reddy VY (2007) SMASH-VT [40] | N/R  (at least one VT zone with ATP as initial therapy) | N/R  (at least one VT zone with ATP as initial therapy) | N/R |
| Sapp JL (2016) VANISH [41] | - 150 bpm - ATPx3 🡪 shock 20-25J 🡪 max shock | - St Jude Medical: 187 bpm - Medtronic: 188-250 bpm (FVT via VF) - Boston/Biotronik: 185 bpm - ATPx1 🡪 max shock | - St Jude Medical/Boston/Biotronik: 250 bpm - Medtronic: 188 bpm - ATP (during charge) 🡪 max shock |
| Žižek d (2024) PREVENTIVE VT [42] | - 10-20 bpm lower than slower VT   Monitor only | - Medtronic: OFF - St Jude Medical: 187 bpm - Biotronik: 188 bpm - Boston/Guidant: 185 bpm - ≥1 ATP (burst of 8 at 84-88% CL) 🡪 shock | - Medtronic: 188 bpm - St Jude Medical: 250 bpm - Biotronik: 231 bpm - Boston/ Guidant: 250 bpm - ATP during charge 🡪 shock |
| Sapp JL (2024) VANISH 2 [43] | - 165-171 bpm & “Monitor only” (if slowest VT>200 bpm) or 20 bpm lower than slowest VT (if VT<200 bpm) or 130 bpm (if VT=140-150 bpm) - ATPx3 🡪 shock 25j 🡪 max shock | - St. Jude Medical: 181-240 bpm - Medtronic (FVT via VF): 182-250 bpm - Boston/Guidant: 180-200 bpm - Biotronik: 190-240 bpm - Microport/Sorin: >230 bpm - ATPx1 🡪 shock (25J-max) | - St. Jude Medical: >240 bpm - Medtronic (FVT via VF): >250 bpm - Boston/Guidant: >200 bpm - Biotronik: >240 bpm - Microport/Sorin: >255 bpm - Max shock (ATP during charge permitted in Medtronic) |
| Seidl K (1998) [44] | N/R | N/R | N/R |
| Pacifico A (1999) Sotalol ICD Study [45] | N/R | N/R | N/R |
| Kettering K (2002) [46] | N/R | N/R | N/R |
| Connolly SJ (2006) OPTIC [47] | No VT-1 zone programmed | - 15-20 bpm lower than slowest VT up to max 170 bpm - ATP 🡪 shock   (St. Jude Medical) | - >222 bpm - Shock only   (St. Jude Medical) |
| Raatikainen P (2025) MANTRA-VT [48] | - 140-170 bpm - Monitor only | - 170-140 bpm or lower if documented VT - ATP 🡪 shock | - >240 bpm - ATP during charge 🡪 shock |
| Abbreviations: ICD, implantable cardioverter-defibrillator; VT, ventricular tachycardia; VF, ventricular fibrillation; bpm, beats per minute; ATP, antitachycardia pacing; CL, cycle length ; FVT, fast VT. | | | |

**Table S.6.** Approaches to data extraction issues

| **Issue** | **Approach** |
| --- | --- |
| Demographic or outcome data were reported separately for the different groups of the same intervention (e.g. different doses) | Groups were merged into a single group using the suggested Cochrane formula [1] |
| Distribution of continuous variables were reported as medians and IQR | Medians and IQR were converted to mean (SD) [2,3] |
| Differences were identified in the reporting of the outcome of HF exacerbation | When data were reported as HF exacerbation or HF hospitalization, these events were preferentially extracted. In case no such events were reported, the events of death due to HF were extracted. |

Abbreviations: LVEF, left ventricular ejection fraction; IQR, interquartile range; HR, hazard ratio; CI, confidence interval; SD, standard deviation.

## **Table S.7.** Extracted variables

First author, study name, year, follow-up, population, type of prevention (primary vs secondary), intervention, arrhythmia-related inclusion criteria, comparator, outcome, number of participants, age, sex, diabetes mellitus, coronary artery disease, myocardial infarction, atrial fibrillation, hypertension, dyslipidaemia, chronic kidney disease, New York Heart Association class, body mass index, left ventricular ejection fraction, renin-angiotensin aldosterone blockers, b-blockers, device programming parameters

## **Table S.8.** Excluded studies with rationale

| **Reason for exclusion (number of studies)** | **Reference** |
| --- | --- |
| Observational/non-randomized (n=6) | [4–9] |
| Ineligible control group (e.g. comparison of different ablation techniques/timing, single-arm studies) (n=6) | [10–15] |
| Ineligible population (e.g. <70% ischemic patients, non-ICD patients) (n=5) | [16–20] |
| Protocol (n=2) | [21,22] |
| Trail completed but no results posted (n=1) | NCT011828839 |
| Ineligible outcomes (n=1) | [23] |
| Ineligible interventions (n=4) | [24–27] |
| Irrelevant sub-analyses of included RCTs (n=7) | [28–35] |
| Abbreviations: ICD, implantable cardioverter defibrillator; RCT, randomized controlled trial. | |

## **Table S.9.** Population characteristics of included records

| First author (Study name), Year | N | | | Age in years, mean (SD) | | Males, n (%) | | DM, n (%) | | CAD, n(%) | | MI, n(%) | | AF, n(%) | | Hypertension, n(%) | | Dyslipidaemia, n(%) | | CKD, n(%) | | NYHA I, n(%) | | NYHA II,n (%) | | NYHA III, n(%) | | NYHA IV, n(%) | | BMI in kg/m2, mean (SD) | | LVEF in %, mean (SD) | | RAAS inhibitor, n(%) | | B-blockers, n(%) | |
| --- | --- | --- | --- | --- | --- | --- | --- | --- | --- | --- | --- | --- | --- | --- | --- | --- | --- | --- | --- | --- | --- | --- | --- | --- | --- | --- | --- | --- | --- | --- | --- | --- | --- | --- | --- | --- | --- |
|  | **Total** | **I** | **C** | **I** | **C** | **I** | **C** | **I** | **C** | **I** | **C** | **I** | **C** | **I** | **C** | **I** | **C** | **I** | **C** | **I** | **C** | **I** | **C** | **I** | **C** | **I** | **C** | **I** | **C** | **I** | **C** | **I** | **C** | **I** | **C** | **I** | **C** |
| Al-Khatib AM (2015) CALYPSO[36] | 25 | 13 | 12 | 62,9 (30,7) | 63,5 (32,9) | 13 (100) | 12 (100) | 6 (46) | 5 (36) | 13 (100) | 12 (100) | 13 (100) | 12 (100) | 5 (39) | 4 (29) | 9 (69) | 12 (86) | 11 (85) | 12 (86) | 2 (15) | 2 (14) | 2 (22) | 3 (21) | 3 (33) | 5 (36) | 1 (11) | 3 (21) | 0 (0) | 0 (0) | N/R | N/R | 36 (41,5) | 36 (41,6) | 11 (85) | 14 (100) | 13 (100) | 12 (86) |
| Arenal A (2022) SURVIVE-VT [37] | 144 | 71 | 73 | 69,3 (9,1) | 70,3 (9) | 70 (98,6) | 68 (93,2) | 21 (29,6) | 15 (20,5) | 71 (100) | 73 (100) | 71 (100) | 73 (100) | 9 (13,6) | 8 (12,3) | 56 (78,9) | 47 (64,4) | N/R | N/R | 8 (11,3) | 7 (9,6) | 31 (44,3) | 31 (42,5) | 33 (47,1) | 37 (50,7) | 6 (8,6) | 5 (6,8) | 0 (0) | 0 (0) | 28,1 (4,9) | 27,8 (3,1) | 31 (43,7) | 36 (49,3) | 70 (98,6) | 65 (90,3) | 69 (97,2) | 62 (89,1) |
| Della Bella P (2023) PARTITA [38] | 45 | 23 | 24 | 71,2 (8,1) | 65,6 (9,6) | 19 (83) | 21 (88) | 4 (19) | 9 (41) | 20 (87) | 18 (75) | N/R | N/R | 9 (39) | 9 (37) | 17 (81) | 15 (68) | 17 (74) | 19 (79) | 3 (14) | 6 (27) | 3 (13) | 5 (24) | 17 (74) | 12 (57) | 3 (13) | 4 (19) | 0 (0) | 0 (0) | N/R | N/R | 31,9 (9) | 32,4 (8,3) | 20 (87) | 22 (92) | 23 (100) | 24 (100) |
| Kuck KH (2010) VTACH [39] | 107 | 52 | 55 | 67,7 (8,3) | 64,4 (8,2) | 50 (96) | 50 (91) | N/R | N/R | 52 (100) | 55 (100) | 52 (100) | 55 (100) | N/R | N/R | N/R | N/R | N/R | N/R | N/R | N/R | N/R | N/R | N/R | N/R | N/R | N/R | N/R | N/R | N/R | N/R | 34 (9,6) | 34,1 (8,8) | N/R | N/R | 39 (75) | 41 (75) |
| Kuck KH (2017) SMS [30] | 111 | 54 | 57 | 68,4 (7,7) | 65,9 (8,4) | 47 (87) | 46 (81) | N/R | N/R | 54 (100) | 57 (100) | 50 (93) | 56 (98) | N/R | N/R | N/R | N/R | N/R | N/R | N/R | N/R | N/R | N/R | N/R | N/R | N/R | N/R | 0 (0) | 0 (0) | N/R | N/R | 32 (6,9) | 30,4 (7,3) | N/R | N/R | 49 (91) | 52 (91) |
| Reddy VY (2007) SMASH-VT [40] | 128 | 64 | 64 | 67 (9) | 66 (10) | 59 (92) | 52 (81) | 24 (38) | 32 (50) | 64 (100) | 64 (100) | 64 (100) | 64 (100) | N/R | N/R | 47 (73) | 43 (67) | 37 (58) | 38 (59) | N/R | N/R | N/R | N/R | N/R | N/R | N/R | N/R | N/R | N/R | N/R | N/R | 30,7 (9,5) | 32,9 (8,5) | 59 (92) | 59 (92) | 60 (94) | 63 (98) |
| Sapp JL (2016) VANISH [41] | 259 | 132 | 127 | 70,3 (7,3) | 67 (8,6) | 118 (92,9) | 123 (93,2) | 40 (31,5) | 37 (28) | 132 (100) | 127 (100) | 132 (100) | 127 (100) | 47 (37) | 52 (39,4) | 88 (69,3) | 92 (69,7) | N/R | N/R | 26 (20,5) | 23 (17,4) | 28 (22) | 33 (25) | 68 (53,5) | 69 (52,3) | 31 (24,4) | 30 (22,7) | 0 (0) | 0 (0) | N/R | N/R | 31,2 (10,7) | 31,1 (10,4) | 111 (87,4) | 116 (88,1) | 122 (96,1) | 124 (93,9) |
| Žižek d (2024) PREVENTIVE VT [42] | 60 | 30 | 30 | 65 (16) | 71 (10) | 29 (96,7) | 26 (86,7) | 8 (26,7) | 9 (30) | 30 (100) | 30 (100) | 30 (100) | 30 (100) | 9 (30) | 10 (33,3) | 24 (80) | 25 (83,3) | N/R | N/R | 6 (20) | 6 (20) | 1 (3,3) | 1 (3,3) | 20 (66,7) | 17 (56,7) | 9 (30) | 12 (40) | 0 (0) | 0 (0) | N/R | N/R | 37 (9) | 34 (8) | 13 (42,3) | 16 (53,3) | 29 (96,7) | 29 (96,7) |
| Sapp JL (2024) VANISH 2 [43] | 416 | 203 | 213 | 67,7 (8,6) | 68,4 (8) | 193 (95,1) | 197 (92,5) | 79 (38,9) | 83 (39) | 203 (100) | 213 (100) | 203 (100) | 213 (100) | 70 (34,5) | 72 (33,8) | 160 (78,8) | 169 (79,3) | N/R | N/R | 31 (15,3) | 23 (10,8) | 87 (42,9) | 89 (41,8) | 99 (48,8) | 107 (50,2) | 17 (8,4) | 17 (8) | 0 (0) | 0 (0) | N/R | N/R | 34 (11) | 34,3 (10,3) | 176 (86,7) | 187 (87,8) | 196 (96,6) | 194 (91,1) |
| Seidl K (1998) [44] | 70 | 35 | 35 | 61 (9) | 62 (10) | 29 (83) | 33 (94,3) | N/R | N/R | 29 (83) | 27 (77,1) | N/R | N/R | N/R | N/R | N/R | N/R | N/R | N/R | N/R | N/R | N/R | N/R | N/R | N/R | N/R | N/R | N/R | N/R | N/R | N/R | 40 (11) | 39 (10) | N/R | N/R | 0 (0) | 35 (100) |
| Pacifico A (1999) Sotalol ICD Study [45] | 302 | 151 | 151 | 63 (11) | 61 (11) | 127 (84) | 124 (82) | N/R | N/R | 110 (73) | 100 (66) | 110 (73) | 100 (66) | N/R | N/R | N/R | N/R | N/R | N/R | N/R | N/R | 87 (58) | 85 (56) | 54 (36) | 60 (40) | 10 (7) | 6 (4) | 0 (0) | 0 (0) | N/R | N/R | 37 (12) | 39 (14) | 64 (42,3) | 54 (35,8) | 27 (17,9) | 28 (18,5) |
| Kettering K (2002) [46] | 100 | 50 | 50 | 59 (12) | 60 (9) | 42 (84) | 41 (82) | N/R | N/R | 35 (70) | 38 (76) | N/R | N/R | N/R | N/R | N/R | N/R | N/R | N/R | N/R | N/R | 10 (20) | 13 (26) | 24 (48) | 20 (40) | 16 (32) | 14 (28) | 0 (0) | 3 (6) | N/R | N/R | 38 (15) | 38 (14) | N/R | N/R | 0 (0) | 100 (0) |
| Connolly SJ (2006) OPTIC [47] | 412 | 274 | 138 | 64,5 (10,1) | 63,2 (9,7) | 228 (83,2) | 115 (83,3) | N/R | N/R | 245 (90,7) | 123 (89,1) | 220 (81,4) | 111 (80,4) | 47 (17,4) | 22 (15,9) | N/R | N/R | N/R | N/R | N/R | N/R | 136 (50,3) | 76 (56) | N/R | N/R | N/R | N/R | 0 (0) | 0 (0) | N/R | N/R | 34 (12 | 34 (11) | N/R | N/R | 140 (51,9) | 138 (100) |
| Raatikainen P (2025) MANTRA-VT [48] | 58 | 28 | 30 | 65,9 | 8,2 | 67,1 | 7,3 | 26 | 93 | 28 | 93 | 9 | 32 | 9 | 30 | 28 | 100 | 30 | 100 | 28 | 100 | 30 | 100 | 6 | 21 | 9 | 30 | 19 | 68 | 17 | 57 | 24 | 85 | 22 | 73 | N/R | N/R |

Abbreviations: SD, standard deviation; LVEF, left ventricular ejection fraction; NYHA, New York Heart Association; HF, Heart failure; I, intervention; C, control

## **Table S.10.** Mapping strategy and procedural endpoints in each VT ablation randomized clinical trial.

| First author (Study name), Year | Arrhythmia related criteria for patient inclusion | Mapping system / strategy | Ablation endpoint |
| --- | --- | --- | --- |
| Al-Khatib AM (2015) CALYPSO [36] | ≥1 appropriate shocks or ≥3 appropriate ATP by the device | The mapping system and method for identifying critical circuit components were left to physicians’ discretion. Activation or substrate mapping based on hemodynamic stability. | Noninducibility of clinical / presumed clinical VT.  Complete noninducibility encouraged, not mandated. |
| Arenal A (2022) SURVIVE-VT [37] | Appropriate shock | Substrate mapping in sinus rhythm. | Complete noninducibility. |
| Della Bella P (2023) PARTITA [38] | First appropriate shock | Hybrid 3D-EAM or non-contact system.  Substrate mapping including EPs, LPs, and LAVAs.  Activation mapping if hemodynamically tolerated VT.  Ablation performed preferably in sinus rhythm. | Complete noninducibility *and* elimination of all LPs. |
| Kuck KH (2010) VTACH [39] | Recorded VT | Hybrid 3D-EAM or non-contact system.  Activation or substrate mapping based on hemodynamic stability. | Complete noninducibility or, if no VT inducible, elimination of channels within area of interest / ablation based on pace mapping. |
| Kuck KH (2017) SMS [30] | Clinically unstable recorded VT or syncope/arrest and inducible VT | Hybrid 3D-EAM or non-contact system or conventional mapping.  Substrate modification targeting LPs – linear lines in good pace mapping matching sites. | Noninducibility of clinical / inducible VT  Complete noninducibility encouraged, not mandated. |
| Reddy VY (2007) SMASH-VT [40] | Recorded VA (VT/VF) – no use of antiarrhythmics | Hybrid 3D-EAM.  Substrate mapping in sinus rhythm, targeting LPs and LAVAs.  Pace mapping in cases of inducibility at initial PVS. | Unclear – supplementary material mentions repeat of PVS at the end of the procedure. |
| Sapp JL (2016) VANISH [41] | ≥1 appropriate shocks or ≥3 appropriate ATP therapies or ≥3 VT within 24 hours (electrical storm) despite AAD | Hybrid 3D-EAM or non-contact system.  Activation /entrainment mapping if VT hemodynamically stable.  Otherwise, substrate mapping including LPs and sites with marked latency (>45msec stimulus to QRS delay). | Complete noninducibility. |
| Žižek d (2024) PREVENTIVE VT [42] | No previous VA (only primary prevention patients) | High density 3D-EAM (mapping and remapping).  Multipolar mapping catheter (minimum of 500 points)  Focus on abnormal decrement-evoked local ventricular EGM | Combined endpoint of abnormal EGM abolition and VT noninducibility with uniform stimulation protocol. |
| Sapp JL (2024) VANISH 2 [43] | Any of:   - Sustained documented monomorphic VT terminated by AAD, DCCV or manual ICD therapy - ≥3 monomorphic VT treated with ATP (at least one symptomatic) - ≥5 monomorphic VT treated with ATP - ≥1 appropriate ICD shocks, - ≥3 monomorphic VT within 24 hours | High-density 3D-EAM  Activation/entrainment/pace mapping first.  Substrate mapping if VT noninducible or very fast (<300 ms) | Noninducibility |
| Raatikainen P (2025) MANTRA-VT [48] | ≥2 documented symptomatic VT/VF (the last being within 12 months) | High-density 3D-EAM  Substrate mapping (targeting fractionated and late potentials in low-voltage areas)  Activation/entrainment/pace mapping if needed. | Noninducibility of clinical and nonclinical VT |
| Abbreviations: VT, ventricular tachycardia; VA, ventricular arrhythmia; VF, ventricular fibrillation; ATP, antitachycardia pacing; ICD, implantable cardioverter-defibrillator; AAD, antiarrhythmic drugs; 3D-EAM, Three-dimensional electroanatomical mapping system; Eps, Early potentials; ILAM, Isochronal latest activation mapping; LAVA, Local abnormal ventricular activities; LPs, Late potentials; PVS, Programmed ventricular stimulation; VT, Ventricular tachycardia. | | | |

## **Table S.11.** Distribution of effect modifiers across different interventions

| **Outcome** | **Intervention** | **Age in years, mean (SD)** | **Percentage of males**  **in %, mean (SD)** | **Percentage of coronary artery disease in %, mean (SD)** | **Percentage of NYHA 1 in %, mean (SD)** | **Left ventricular ejection fraction in %, mean (SD)** | **Beta blockers’ usage in %, mean (SD)** |
| --- | --- | --- | --- | --- | --- | --- | --- |
| **A. Efficacy outcomes** | | | | | | | |
| Appropriate shock | CA | 68 (1.3) | 91 (4.2) | 98 (5.5) | 68 (1.3) | 33 (1.2) | 93 (8.5) |
|  | Class III AAD | 67 (2.2) | 92 (4.9) | 98 (4.8) | 67 (2.2) | 33 (1.3) | 89 (12.4) |
|  | SC | 66 (1.5) | 87 (4.2) | 95 (7.4) | 66 (1.5) | 33 (1.3) | 87 (15.3) |
| Electrical storm | CA | 68 (1.3) | 91 (4.2) | 98 (5.5) | 68 (1.3) | 33 (1.2) | 93 (8.5) |
|  | Class III AAD | 68 (1.4) | 94 (1.3) | 100 (0) | 68 (1.4) | 33 (1.4) | 95 (3) |
|  | SC | 67 (1) | 88 (4.1) | 96 (7.8) | 67 (1) | 32 (1.3) | 92 (12.5) |
| Ventricular arrhythmia recurrence | CA | 67 (2) | 92 (5.2) | 98 (5.5) | 67 (2) | 33 (1.6) | 94 (8.9) |
|  | Class III AAD | 67 (2.8) | 96 (3.1) | 100 (0) | 67 (2.8) | 34 (1.6) | 96 (4.1) |
|  | SC | 67 (1) | 88 (4.1) | 96 (7.8) | 67 (1) | 32 (1.3) | 92 (12.5) |
| All-cause mortality | CA | 67 (1.9) | 92 (4.9) | 98 (5.2) | 67 (1.9) | 33 (1.6) | 94 (8.3) |
|  | Class III AAD | 67 (2.6) | 93 (5.5) | 98 (4.4) | 67 (2.6) | 34 (1.7) | 91 (11.9) |
|  | SC | 66 (1.5) | 87 (4.2) | 95 (7.4) | 66 (1.5) | 33 (1.3) | 87 (15.3) |
| Cardiovascular mortality | CA | 68 (1) | 91 (4.8) | 97 (7) | 68 (1) | 32 (1.4) | 95 (5.5) |
|  | Class III AAD | 69 (0.9) | 94 (1.4) | 100 (0) | 69 (0.9) | 33 (1.6) | 93 (2.1) |
|  | SC | 68 (0.8) | 86 (3.6) | 92 (11) | 68 (0.8) | 32 (0.7) | 98 (9.5) |
| Heart failure exacerbation | CA | 67 (2.2) | 93 (4.4) | 98 (5.9) | 67 (2.2) | 33 (1.7) | 97 (4.4) |
|  | Class III AAD | 67 (2.6) | 93 (5.5) | 98 (4.4) | 67 (2.6) | 34 (1.7) | 91 (11.9) |
|  | SC | 66 (2.1) | 86 (2.8) | 91 (8) | 66 (2.1) | 33 (1.2) | 89 (19.4) |
| Undetected VT | SC | 66 (2.1) | 86 (2.8) | 91 (8) | 66 (2.1) | 33 (1.2) | 89 (19.4) |
|  | CA | 69 (0.9) | 94 (1.4) | 100 (0) | 69 (0.9) | 33 (1.6) | 93 (2.1) |
|  | Class III AAD | 69 (0.9) | 94 (1.4) | 100 (0) | 69 (0.9) | 33 (1.6) | 93 (2.1) |
| Cardiac hospitalization | CA | 68 (1.6) | 94 (1.2) | 100 (0) | 68 (1.6) | 33 (1.4) | 89 (9.4) |
|  | Class III AAD | 69 (0.9) | 94 (1.4) | 100 (0) | 69 (0.9) | 33 (1.6) | 93 (2.1) |
|  | SC | 66 (NA) | 94 (NA) | 100 (NA) | 66 (NA) | 34 (NA) | 75 (NA) |
| Ventricular arrhythmia hospitalization | CA | 66 (3.3) | 96 (3.5) | 100 (0) | 66 (3.3) | 34 (2) | 96 (4.8) |
|  | Class III AAD | 66 (3.3) | 96 (3.5) | 100 (0) | 66 (3.3) | 34 (2) | 96 (4.8) |
|  | CA | 68 (1.2) | 93 (2.5) | 97 (7) | 68 (1.2) | 33 (1.2) | 96 (5.1) |
| Appropriate ATP | Class III AAD | 68 (1.4) | 94 (1.3) | 100 (0) | 68 (1.4) | 33 (1.4) | 95 (3) |
|  | SC | 68 (NA) | 89 (NA) | 84 (NA) | 68 (NA) | 32 (NA) | 104 (NA) |
|  | CA | 68 (1.3) | 91 (4.2) | 98 (5.5) | 68 (1.3) | 33 (1.2) | 93 (8.5) |
| **B. Safety outcomes** | | | | | | | |
| Any serious AE | CA | 68 (0.4) | 93 (0.5) | 100 (0) | 68 (0.4) | 33 (2.1) | 94 (0.8) |
|  | Class III AAD | 68 (0.4) | 93 (0.5) | 100 (0) | 68 (0.4) | 33 (2.1) | 94 (0.8) |
|  | CA | 68 (1.9) | 90 (8.5) | 100 (0) | 68 (1.9) | 32 (1.6) | 91 (0) |
| Cardiac perforation | Class III AAD | 70 (NA) | 96 (NA) | 100 (NA) | 70 (NA) | 34 (NA) | 91 (NA) |
|  | SC | 67 (NA) | 84 (NA) | 100 (NA) | 67 (NA) | 31 (NA) | 91 (NA) |
| Pericardial effusion (drainage) | CA | 68 (1.1) | 92 (5.3) | 100 (0) | 68 (1.1) | 33 (1.6) | 93 (2) |
|  | Class III AAD | 69 (0.9) | 94 (1.4) | 100 (0) | 69 (0.9) | 33 (1.6) | 93 (2.1) |
|  | SC | 67 (NA) | 84 (NA) | 100 (NA) | 67 (NA) | 31 (NA) | 91 (NA) |
| Stroke/TIA | CA | 68 (0.4) | 93 (0.5) | 100 (0) | 68 (0.4) | 33 (2.1) | 94 (0.8) |
|  | Class III AAD | 68 (0.4) | 93 (0.5) | 100 (0) | 68 (0.4) | 33 (2.1) | 94 (0.8) |
|  | CA | 67 (3) | 96 (3.8) | 100 (0) | 67 (3) | 34 (2.4) | 96 (3.3) |
| Major bleeding | Class III AAD | 66 (2.8) | 93 (6.9) | 97 (5.4) | 66 (2.8) | 34 (2) | 89 (14.6) |
|  | SC | 64 (NA) | 83 (NA) | 89 (NA) | 64 (NA) | 34 (NA) | 68 (NA) |
| Myocardial infarction | CA | 68 (1.4) | 94 (1.3) | 100 (0) | 68 (1.4) | 33 (1.4) | 95 (3) |
|  | Class III AAD | 67 (2.2) | 92 (4.9) | 98 (4.8) | 67 (2.2) | 33 (1.3) | 89 (12.4) |
|  | SC | 64 (NA) | 83 (NA) | 89 (NA) | 64 (NA) | 34 (NA) | 68 (NA) |
| Hypothyroidism | CA | 69 (0.9) | 94 (1.4) | 100 (0) | 69 (0.9) | 33 (1.6) | 93 (2.1) |
|  | Class III AAD | 68 (2.5) | 92 (5.6) | 97 (5.4) | 68 (2.5) | 33 (1.4) | 87 (13) |
|  | SC | 64 (NA) | 83 (NA) | 89 (NA) | 64 (NA) | 34 (NA) | 68 (NA) |
| Hyperthyroidism | CA | 68 (0.4) | 93 (0.5) | 100 (0) | 68 (0.4) | 33 (2.1) | 94 (0.8) |
|  | Class III AAD | 68 (0.4) | 93 (0.5) | 100 (0) | 68 (0.4) | 33 (2.1) | 94 (0.8) |
|  | CA | 68 (1.9) | 90 (8.5) | 100 (0) | 68 (1.9) | 32 (1.6) | 91 (0) |
| Pulmonary toxicity | CA | 68 (1.4) | 94 (1.3) | 100 (0) | 68 (1.4) | 33 (1.4) | 95 (3) |
|  | Class III AAD | 67 (2.2) | 92 (4.9) | 98 (4.8) | 67 (2.2) | 33 (1.3) | 89 (12.4) |
|  | SC | 64 (NA) | 83 (NA) | 89 (NA) | 64 (NA) | 34 (NA) | 68 (NA) |
| Liver toxicity | CA | 68 (0.4) | 93 (0.5) | 100 (0) | 68 (0.4) | 33 (2.1) | 94 (0.8) |
|  | Class III AAD | 68 (0.4) | 93 (0.5) | 100 (0) | 68 (0.4) | 33 (2.1) | 94 (0.8) |

Abbreviations: CA, catheter ablation; AAD, antiarrhythmic drugs; SC, standard care; NA, not applicable; SD, standard deviation; NYHA, New York Heart Association; ATP, antitachycardia pacing; AE, adverse event; TIA, transient ischemic attack

## **Table S.12.** Distribution of effect modifiers across different comparisons

| **Comparison** | **Age in years, mean (SD)** | **Percentage of males**  **in %, mean** | **Percentage of coronary artery disease in %, mean** | **Percentage of NYHA 1 in %, mean** | **Left ventricular ejection fraction in %, mean** | **Beta blockers’ usage in %, mean** |
| --- | --- | --- | --- | --- | --- | --- |
| A. **Efficacy outcomes** | | | | | | |
| **Appropriate shock** | | | | | | |
| CA:Class III AAD | 68.3 | 93.9 | 100 | 68.3 | 32.8 | 94.5 |
| CA:SC | 67 | 88.2 | 96.1 | 67 | 32.3 | 91.6 |
| Class III AAD:SC | 61.8 | 84.5 | 78 | 61.8 | 37.4 | 58.9 |
| **Electrical storm** | | | | | | |
| CA:Class III AAD | 68.3 | 93.9 | 100 | 68.3 | 32.8 | 94.5 |
| CA:SC | 67 | 88.2 | 96.1 | 67 | 32.3 | 91.6 |
| **VA recurrence** | | | | | | |
| CA:Class III AAD | 66.9 | 95.7 | 100 | 66.9 | 34 | 95.8 |
| CA:SC | 67 | 88.2 | 96.1 | 67 | 32.3 | 91.6 |
| Class III AAD:SC | 60.5 | 85.8 | 76.5 | 60.5 | 38.8 | 75 |
| **All-cause mortality** | | | | | | |
| CA:Class III AAD | 67.3 | 95.2 | 100 | 67.3 | 33.4 | 95.6 |
| CA:SC | 67 | 88.2 | 96.1 | 67 | 32.3 | 91.6 |
| **Cardiovascular mortality** | | | | | | |
| CA:Class III AAD | 68.9 | 94.2 | 100 | 68.9 | 33 | 93.3 |
| CA:SC | 67.7 | 86.3 | 92.2 | 67.7 | 31.7 | 97.7 |
| **Heart failure exacerbation** | | | | | | |
| CA:Class III AAD | 67.3 | 95.2 | 100 | 67.3 | 33.4 | 95.6 |
| CA:SC | 67.4 | 87.8 | 92.2 | 67.4 | 32 | 100.2 |
| Class III AAD:SC | 61.9 | 83.1 | 77.3 | 61.9 | 36.7 | 61.9 |
| **Undetected VT** | | | | | | |
| CA:Class III AAD | 68.9 | 94.2 | 100 | 68.9 | 33 | 93.3 |
| **Cardiac hospitalization** | | | | | | |
| CA:Class III AAD | 68.9 | 94.2 | 100 | 68.9 | 33 | 93.3 |
| CA:SC | 66 | 93.5 | 100 | 66 | 34.1 | 74.8 |
| **Ventricular arrhythmia hospitalization** | | | | | | |
| CA:Class III AAD | 66.5 | 96.3 | 100 | 66.5 | 33.9 | 96.4 |
| **Appropriate ATP** | | | | | | |
| CA:Class III AAD | 68.3 | 93.9 | 100 | 68.3 | 32.8 | 94.5 |
| CA:SC | 68.3 | 88.9 | 84.4 | 68.3 | 32.2 | 104.4 |
| B. **Safety outcomes** | | | | | | |
| **Any serious AE** | | | | | | |
| CA:Class III AAD | 67 | 96.5 | 100 | 67 | 34.6 | 94.9 |
| CA:SC | 67.1 | 83.8 | 100 | 67.1 | 31.2 | 91 |
| **Cardiac perforation** | | | | | | |
| CA:Class III AAD | 68.4 | 93.4 | 100 | 68.4 | 32.7 | 94.4 |
| **Pericardial effusion (drainage)** | | | | | | |
| CA:Class III AAD | 69.8 | 95.8 | 100 | 69.8 | 33.5 | 91 |
| CA:SC | 67.1 | 83.8 | 100 | 67.1 | 31.2 | 91 |
| **Stroke or TIA** | | | | | | |
| CA:Class III AAD | 68.9 | 94.2 | 100 | 68.9 | 33 | 93.3 |
| CA:SC | 67.1 | 83.8 | 100 | 67.1 | 31.2 | 91 |
| Class III AAD:SC | 59.5 | 83 | 73 | 59.5 | 38 | 100 |
| **Major bleeding** | | | | | | |
| CA:Class III AAD | 68.4 | 93.4 | 100 | 68.4 | 32.7 | 94.4 |
| **Myocardial infarction** | | | | | | |
| CA:Class III AAD | 66.7 | 95.6 | 100 | 66.7 | 33.8 | 96.3 |
| Class III AAD:SC | 61.9 | 83.1 | 77.3 | 61.9 | 36.7 | 61.9 |
| **Hypothyroidism** | | | | | | |
| CA:Class III AAD | 68.3 | 93.9 | 100 | 68.3 | 32.8 | 94.5 |
| Class III AAD:SC | 64.1 | 83.3 | 89.3 | 64.1 | 34 | 67.5 |
| **Hyperthyroidism** | | | | | | |
| CA:Class III AAD | 68.9 | 94.2 | 100 | 68.9 | 33 | 93.3 |
| Class III AAD:SC | 64.1 | 83.3 | 89.3 | 64.1 | 34 | 67.5 |
| **Pulmonary toxicity** | | | | | | |
| CA:Class III AAD | 68.3 | 93.9 | 100 | 68.3 | 32.8 | 94.5 |
| Class III AAD:SC | 64.1 | 83.3 | 89.3 | 64.1 | 34 | 67.5 |
| **Liver toxicity** | | | | | | |
| CA:Class III AAD | 68.4 | 93.4 | 100 | 68.4 | 32.7 | 94.4 |

Abbreviations: CA, catheter ablation; AAD, antiarrhythmic drugs; SC, standard care; NA, not applicable; SD, standard deviation; NYHA, New York Heart Association; ATP, antitachycardia pacing; AE, adverse event; TIA, transient ischemic attack

## **Table S.13**. Detailed methodological quality assessments of included studies based on the Cochrane Risk of Bias v.2 tool

| **First author, Year** | **Domain 1. Randomization process** | **Domain 2. Deviations from intended interventions** | **Domain 3. Mising outcome data** | **Domain 4. Measurement of the outcome** | **Domain 5. Selection of the reported result** | **Domain 6. Overall Bias** |
| --- | --- | --- | --- | --- | --- | --- |
| Al-Khatib AM (2015) CALYPSO[36] | High | Some Concerns | Low | Low | Low | High |
| Arenal A (2022) SURVIVE-VT [37] | Low | Low | Low | Low | Low | Low |
| Della Bella P (2023) PARTITA [38] | High | Some Concerns | Low | Some Concerns | Low | High |
| Kuck KH (2010) VTACH [39] | Low | Some Concerns | Low | Low | Low | Some Concerns |
| Kuck KH (2017) SMS [30] | Low | Low | Low | Low | Low | Low |
| Reddy VY (2007) SMASH-VT [40] | Some Concerns | Some Concerns | Low | Some Concerns | Low | Some Concerns |
| Sapp JL (2016) VANISH [41] | Low | Low | Low | Low | Low | Low |
| Žižek d (2024) PREVENTIVE VT [42] | Low | Low | Low | Low | Low | Low |
| Sapp JL (2024) VANISH 2 [43] | Low | Low | Low | Low | Low | Low |
| Seidl K (1998) [44] | Some Concerns | Low | Low | Some Concerns | Low | Some Concerns |
| Pacifico A (1999) Sotalol ICD Study [45] | Some Concerns | Low | Low | Low | Low | Low |
| Kettering K (2002) [46] | Some Concerns | Low | Low | Some Concerns | Low | Some Concerns |
| Connolly SJ (2006) OPTIC [47] | Low | Low | Low | Low | Low | Low |
| Raatikainen P (2025) MANTRA-VT [48] | Low | Low | Low | Low | Low | Low |

## **Table S.14**. League tables for main analysis

| A. **Efficacy outcomes (RR with 95% CI)** | | |
| --- | --- | --- |
| **Appropriate shock** | | |
| CA | 0.76 [0.49; 1.19] | 0.44 [0.26; 0.75] |
| 0.68 [0.47; 0.99] | Class III AAD | 0.86 [0.56; 1.33] |
| 0.52 [0.35; 0.78] | 0.77 [0.53; 1.11] | SC |
| **Electrical storm** | | |
| CA | 0.81 [0.63; 1.03] | 0.62 [0.39; 1.01] |
| 0.81 [0.63; 1.03] | Class III AAD | . |
| 0.62 [0.39; 1.01] | 0.77 [0.45; 1.32] | SC |
| **Ventricular arrhythmia recurrence** | | |
| CA | 0.95 [0.73; 1.23] | 0.71 [0.54; 0.95] |
| 0.86 [0.68; 1.08] | Class III AAD | 1.12 [0.77; 1.63] |
| 0.80 [0.63; 1.02] | 0.93 [0.71; 1.21] | SC |
| **All-cause mortality** | | |
| CA | 0.92 [0.72; 1.19] | 0.67 [0.39; 1.15] |
| 0.88 [0.70; 1.12] | Class III AAD | 1.18 [0.65; 2.13] |
| 0.82 [0.54; 1.23] | 0.92 [0.61; 1.41] | SC |
| **Cardiovascular mortality** | | |
| CA | 1.04 [0.74; 1.47] | 0.56 [0.18; 1.71] |
| 1.04 [0.74; 1.47] | Class III AAD | . |
| 0.56 [0.18; 1.71] | 0.54 [0.17; 1.73] | SC |
| **Heart failure exacerbation** | | |
| CA | 0.79 [0.60; 1.05] | 0.41 [0.13; 1.29] |
| 0.75 [0.57; 0.99] | Class III AAD | 1.48 [0.92; 2.39] |
| 0.96 [0.58; 1.58] | 1.27 [0.82; 1.99] | SC |
| **Undetected VT** | | |
| CA | 0.27 [0.15; 0.46] |  |
| 0.27 [0.15; 0.46] | Class III AAD |  |
| **Cardiac hospitalization** | | |
| CA | 0.79 [0.57; 1.09] | 0.60 [0.32; 1.12] |
| 0.79 [0.57; 1.09] | Class III AAD | . |
| **Ventricular arrhythmia hospitalization** | | |
| CA | 0.66 [0.22; 1.98] |  |
| 0.66 [0.22; 1.98] | Class III AAD |  |
| **Appropriate ATP** | | |
| CA | 0.97 [0.85; 1.10] | 0.66 [0.31; 1.41] |
| 0.96 [0.85; 1.10] | Class III AAD | 1.00 [0.53; 1.87] |
| 0.83 [0.51; 1.35] | 0.86 [0.53; 1.40] | SC |
| B. **Safety outcomes (RR with 95% CI)** | | |
| **Any serious AE** | | |
| CA | 0.63 [0.33; 1.23] | 1.09 [0.43; 2.78] |
| 0.63 [0.33; 1.23] | Class III AAD | . |
| 1.09 [0.43; 2.78] | 1.72 [0.54; 5.42] | SC |
| **Cardiac perforation** | | |
| CA | 2.83 [0.43; 18.44] |  |
| 2.83 [0.43; 18.44] | Class III AAD |  |
| **Pericardial effusion (pericardiocentesis)** | | |
| CA | 3.08 [0.13; 74.46] | 5.28 [0.26; 107.41] |
| 3.08 [0.13; 74.46] | Class III AAD | . |
| 5.28 [0.26; 107.41] | 1.71 [0.02; 137.13] | SC |
| **Stroke or TIA** | | |
| CA | 1.94 [0.81; 4.67] | 5.28 [0.26; 107.41] |
| 1.94 [0.81; 4.67] | Class III AAD | . |
| 5.28 [0.26; 107.41] | 2.72 [0.12; 62.74] | SC |
| **Major bleeding** | | |
| CA | 2.42 [0.75; 7.81] |  |
| 2.42 [0.75; 7.81] | Class III AAD |  |
| **Myocardial infarction** | | |
| CA | 1.39 [0.59; 3.24] | . |
| 1.39 [0.59; 3.24] | Class III AAD | 0.39 [0.07; 2.25] |
| 0.54 [0.08; 3.80] | 0.39 [0.07; 2.25] | SC |
| **Hypothyroidism** | | |
| CA | 0.49 [0.21; 1.12] | . |
| 0.49 [0.21; 1.12] | Class III AAD | 7.57 [0.44; 131.55] |
| 3.69 [0.19; 72.15] | 7.57 [0.44; 131.55] | SC |
| **Hyperthyroidism** | | |
| CA | 0.40 [0.17; 0.92] | . |
| 0.40 [0.17; 0.92] | Class III AAD | 2.52 [0.12; 52.19] |
| 1.00 [0.04; 23.29] | 2.52 [0.12; 52.19] | SC |
| **Pulmonary toxicity** | | |
| CA | 0.18 [0.03; 1.02] | . |
| 0.18 [0.03; 1.02] | Class III AAD | 11.60 [0.69; 195.48] |
| 2.11 [0.08; 57.61] | 11.60 [0.69; 195.48] | SC |
| **Liver toxicity** | | |
| CA | 0.28 [0.08; 1.04] |  |
| 0.28 [0.08; 1.04] | Class III AAD |  |

For all outcomes, the effect estimates on the upper right part of the diagonal represent the direct effect estimates while the lower left part represents the combined network estimates. Abbreviations: RR, risk ratio; CI, confidence interval; CA, catheter ablation; AAD, antiarrhythmic drugs; SC, standard care; NYHA, New York Heart Association; ATP, antitachycardia pacing; AE, adverse event; TIA, transient ischemic attack.

## **Table S.15**. Characteristics of outcome networks in the secondary analysis (splitting Class III AAD)

| **Outcome** | **Studies (n)** | **Interventions**  **(n)** | **I^2^ (95% CI)** | **p_heterogeneity_** | **p_inconsistency_** |
| --- | --- | --- | --- | --- | --- |
| Appropriate shock | 12 | 4 | 0.59 (0.25, 0.78) | 0.001 | 0.657 |
| Electrical storm | 8 | 4 | 0.23 (0, 0.66) | 0.213 | 0.393 |
| VA recurrence | 8 | 4 | 0.57 (0, 0.82) | 0.042 | N/A |
| All-cause mortality | 12 | 4 | 0.32 (0, 0.66) | 0.341 | 0.081 |
| CV mortality | 5 | 4 | 0.59 (0, 0.85) | 0.02 | 0.884 |
| HF exacerbation | 9 | 4 | 0.44 (0, 0.73) | 0.142 | 0.095 |
| Undetected VT | 3 | 3 | 0 (0, 0.9) | 0.698 | 0.883 |
| Cardiac hospitalization | 3 | 3 | 0.49 (N/A) | 0.162 | N/A |
| VA hospitalization | 2 | 2 | 0.8 (0.14, 0.95) | N/A | N/A |
| Appropriate ATP | 5 | 4 | 0.6 (0, 0.89) | 0.081 | N/A |
| Any serious AE | 2 | 4 | N/A | N/A | N/A |
| Pericardial effusion (pericardiocentesis) | 2 | 3 | N/A | N/A | N/A |
| Stroke or TIA | 5 | 4 | 0 (0, 0.85) | 0.617 | 0.768 |
| Major bleeding | 2 | 3 | 0.42 (N/A) | N/A | 0.189 |
| Myocardial infarction | 5 | 4 | 0 (0, 0.79) | 0.269 | 0.904 |
| Hypothyroidism | 4 | 4 | 0 (0, 0.9) | 0.998 | N/A |
| Pulmonary toxicity | 5 | 4 | 0 (0, 0.79) | 0.989 | 0.459 |
| Liver toxicity | 1 | 2 | N/A | N/A | N/A |

Abbreviations: N/A, not applicable; CI, confidence interval; VA, ventricular arrhythmia; CV, cardiovascular; HF, heart failure; VT, ventricular tachycardia; ATP, antitachycardia pacing; AE, adverse event; TIA, transient ischemic attack.

## **Table S.16**. League tables for secondary analysis (splitting Class III AAD)

| A. **Efficacy outcomes (RR with 95% CI)** | | | |
| --- | --- | --- | --- |
| **Appropriate shock** | | | |
| CA | 0.88 [0.53; 1.48] | 0.47 [0.24; 0.93] | 0.43 [0.24; 0.78] |
| 0.91 [0.57; 1.46] | Amiodarone | 0.43 [0.23; 0.79] | 0.32 [0.10; 0.96] |
| 0.42 [0.26; 0.69] | 0.47 [0.27; 0.79] | Sotalol | 0.95 [0.58; 1.56] |
| 0.41 [0.26; 0.65] | 0.45 [0.26; 0.78] | 0.97 [0.63; 1.48] | SC |
| **Electrical storm** | | | |
| CA | 0.94 [0.61; 1.45] | 0.79 [0.60; 1.02] | 0.62 [0.39; 1.01] |
| 0.89 [0.58; 1.35] | Sotalol | 0.97 [0.60; 1.57] | . |
| 0.79 [0.60; 1.02] | 0.88 [0.57; 1.37] | Amiodarone | . |
| 0.62 [0.39; 1.01] | 0.70 [0.37; 1.33] | 0.79 [0.46; 1.37] | SC |
| **Ventricular arrhythmia recurrence** | | | |
| CA | 0.74 [0.37; 1.46] | 0.68 [0.44; 1.04] | . |
| 0.74 [0.37; 1.46] | Amiodarone | . | . |
| 0.68 [0.44; 1.04] | 0.92 [0.41; 2.06] | SC | 0.78 [0.43; 1.41] |
| 0.53 [0.25; 1.10] | 0.72 [0.26; 1.95] | 0.78 [0.43; 1.41] | Sotalol |
| **All-cause mortality** | | | |
| Sotalol | 0.65 [0.31; 1.36] | 1.14 [0.59; 2.20] | 0.46 [0.24; 0.87] |
| 0.85 [0.50; 1.44] | CA | 0.65 [0.35; 1.20] | 0.79 [0.52; 1.20] |
| 0.78 [0.46; 1.34] | 0.92 [0.56; 1.52] | SC | 0.34 [0.06; 1.78] |
| 0.61 [0.35; 1.06] | 0.72 [0.49; 1.07] | 0.79 [0.44; 1.40] | Amiodarone |
| **Cardiovascular mortality** | | | |
| CA | 1.02 [0.23; 4.60] | 1.03 [0.32; 3.30] | 0.46 [0.08; 2.74] |
| 0.98 [0.23; 4.15] | Sotalol | 0.96 [0.19; 4.81] | . |
| 0.96 [0.30; 3.07] | 0.98 [0.22; 4.42] | Amiodarone | . |
| 0.46 [0.08; 2.74] | 0.47 [0.05; 4.66] | 0.47 [0.06; 4.02] | SC |
| **Heart failure exacerbation** | | | |
| CA | 0.41 [0.11; 1.45] | 1.10 [0.48; 2.49] | 0.69 [0.41; 1.17] |
| 0.84 [0.40; 1.73] | SC | 0.76 [0.38; 1.51] | 0.76 [0.25; 2.35] |
| 0.82 [0.43; 1.55] | 0.98 [0.53; 1.82] | Sotalol | 0.73 [0.38; 1.39] |
| 0.67 [0.41; 1.11] | 0.80 [0.39; 1.65] | 0.82 [0.45; 1.49] | Amiodarone |
| **Undetected VT** | | | |
| CA | 0.27 [0.15; 0.49] | 0.26 [0.12; 0.55] |  |
| 0.27 [0.15; 0.49] | Amiodarone | 0.90 [0.49; 1.65] |  |
| 0.25 [0.13; 0.49] | 0.91 [0.51; 1.64] | Sotalol |  |
| **Cardiac hospitalization** | | | |
| CA | 0.66 [0.41; 1.07] | 0.60 [0.31; 1.17] |  |
| 0.66 [0.41; 1.07] | Amiodarone | . |  |
| 0.60 [0.31; 1.17] | 0.90 [0.40; 2.05] | SC |  |
| **Ventricular arrhythmia hospitalization** | | | |
| CA | 0.67 [0.08; 5.26] |  |  |
| 0.67 [0.08; 5.26] | Amiodarone |  |  |
|  |  |  |  |
| **Appropriate ATP** | | | |
| CA | . | 0.72 [0.38; 1.36] | 0.66 [0.21; 2.10] |
| 0.66 [0.14; 3.19] | Sotalol | . | 1.00 [0.34; 2.92] |
| 0.72 [0.38; 1.36] | 1.08 [0.20; 5.88] | Amiodarone | . |
| 0.66 [0.21; 2.10] | 1.00 [0.34; 2.92] | 0.93 [0.25; 3.46] | SC |
| B. **Safety outcomes (RR with 95% CI)** | | | |
| **Any serious AE** | | | |
| SC | 0.92 [0.68; 1.24] | . | . |
| 0.92 [0.68; 1.24] | CA | 0.39 [0.12; 1.32] | 0.33 [0.15; 0.75] |
| 0.36 [0.10; 1.26] | 0.39 [0.12; 1.32] | Sotalol | 0.85 [0.30; 2.43] |
| 0.31 [0.13; 0.72] | 0.33 [0.15; 0.75] | 0.85 [0.30; 2.43] | Amiodarone |
| **Pericardial effusion (drainage)** | | | |
| SC | . | 0.19 [0.01; 3.86] |  |
| 0.58 [0.01; 46.86] | Amiodarone | 0.32 [0.01; 7.83] |  |
| 0.19 [0.01; 3.86] | 0.32 [0.01; 7.83] | CA |  |
|  |  |  |  |
| **Stroke or TIA** | | | |
| SC | 1.00 [0.06; 15.55] | . | 0.19 [0.01; 3.86] |
| 0.77 [0.09; 6.42] | Sotalol | 0.52 [0.10; 2.80] | 0.39 [0.09; 1.75] |
| 0.43 [0.04; 4.40] | 0.56 [0.12; 2.68] | Amiodarone | 0.61 [0.23; 1.61] |
| 0.26 [0.03; 2.29] | 0.34 [0.08; 1.38] | 0.61 [0.23; 1.60] | CA |
| **Major bleeding** | | | |
| Sotalol | 0.21 [0.01; 5.74] | 0.28 [0.01; 7.15] |  |
| 0.32 [0.01; 7.87] | Amiodarone | 0.62 [0.14; 2.68] |  |
| 0.20 [0.01; 4.71] | 0.62 [0.14; 2.68] | CA |  |
| **Myocardial infarction** | | | |
| Sotalol | 0.84 [0.21; 3.47] | 0.58 [0.12; 2.87] | 0.59 [0.16; 2.08] |
| 0.78 [0.22; 2.76] | Amiodarone | 0.99 [0.10; 9.36] | 0.67 [0.23; 1.95] |
| 0.67 [0.16; 2.82] | 0.85 [0.17; 4.35] | SC | . |
| 0.56 [0.17; 1.79] | 0.71 [0.26; 1.98] | 0.84 [0.16; 4.49] | CA |
| **Hypothyroidism** | | | |
| SC | 0.32 [0.01; 7.88] | . | 0.08 [0.00; 1.37] |
| 0.32 [0.01; 7.88] | Sotalol | . | 0.24 [0.04; 1.40] |
| 0.21 [0.01; 4.91] | 0.65 [0.07; 5.82] | CA | 0.37 [0.10; 1.39] |
| 0.08 [0.00; 1.37] | 0.24 [0.04; 1.40] | 0.37 [0.10; 1.39] | Amiodarone |
| **Pulmonary toxicity** | | | |
| SC | . | 0.11 [0.01; 1.98] | 0.07 [0.00; 1.17] |
| 0.26 [0.01; 5.58] | CA | 2.57 [0.12; 52.99] | 0.22 [0.07; 0.66] |
| 0.13 [0.01; 2.32] | 0.48 [0.11; 2.18] | Sotalol | 0.48 [0.17; 1.40] |
| 0.06 [0.00; 1.06] | 0.23 [0.08; 0.71] | 0.48 [0.17; 1.39] | Amiodarone |
| **Liver toxicity** | | | |
| CA | 0.07 [0.00; 1.30] |  |  |
| 0.07 [0.00; 1.30] | Amiodarone |  |  |
|  |  |  |  |

For all outcomes, the effect estimates on the upper right part of the diagonal represent the direct effect estimates while the lower left part represents the combined network estimates. Abbreviations: RR, risk ratio; CI, confidence interval; CA, catheter ablation; AAD, antiarrhythmic drugs; SC, standard care; NYHA, New York Heart Association; ATP, antitachycardia pacing; AE, adverse event; TIA, transient ischemic attack.

## **Table S.17**. Studies included in each analysis

| **First author (Study name), Year** | **Main analysis** | **Secondary analysis (splitting AAD)** | **Primary and secondary prevention** | **CAD only** | **Studies including ≥100 patients** | **Studies enrolling after year 2000** | **Studies enrolling after the publication of MADIT-RIT trial** |
| --- | --- | --- | --- | --- | --- | --- | --- |
| **Al-Khatib AM (2015) CALYPSO[36]** | **X** | **-** | **X** | **X** | **-** | **X** | **X** |
| **Arenal A (2022) SURVIVE-VT [37]** | **X** | **X** | **X** | **X** | **X** | **X** | **X** |
| **Della Bella P (2023) PARTITA [38]** | **X** | **X** | **X** | **-** | **-** | **X** | **X** |
| **Kuck KH (2010) VTACH [39]** | **X** | **X** | **X** | **X** | **X** | **X** | **-** |
| **Kuck KH (2017) SMS [30]** | **X** | **X** | **X** | **X** | **X** | **X** | **X** |
| **Reddy VY (2007) SMASH-VT [40]** | **X** | **X** | **X** | **X** | **X** | **X** | **-** |
| **Sapp JLX (2016) VANISH [41]** | **X** | **X** | **X** | **X** | **X** | **X** | **X** |
| **Žižek d (2024) PREVENTIVE VT [42]** | **-** | **-** | **X** | **-** | **-** | **-** | **-** |
| **Sapp JL (2024) VANISH 2 [43]** | **X** | **X** | **X** | **X** | **X** | **X** | **X** |
| **Seidl K (1998) [73]** | **X** | **X** | **X** | **-** | **-** | **-** | **-** |
| **Pacifico A (1999) Sotalol ICD Study [74]** | **X** | **X** | **X** | **-** | **X** | **-** | **-** |
| **Kettering K (2002) [75]** | **X** | **X** | **X** | **-** | **X** | **-** | **-** |
| **Connolly SJ (2006) OPTIC [9]** | **X** | **X** | **X** | **-** | **X** | **X** | **-** |
| **Raatikainen P (2025) MANTRA-VT [48]** | **X** | **X** | **X** | **X** | **-** | **X** | **X** |

Abbreviations: AAD, antiarrhythmic drugs; CAD, coronary artery disease.

Studies included in review

Main analysis (n = 13)

Sensitivity analyses (n = 14)

**Identification**

**Included**

Records screened

(n = 10259)

Records excluded

(n =10213)

Reports sought for retrieval

(n = 46)

Reports not retrieved

(n = 0)

Reports assessed for eligibility

(n = 46)

**Screening**

Records identified from*:

MEDLINE (n = 8225)

SCOPUS (n = 10169)

Cochrane Library (n=2161)

ClinicalTrials.gov (n=1)

Other sources (n=0)

Records removed *before screening*:

Duplicate records removed (n = 10297)

Reports excluded (n=32):

- Observational/non-randomized (n=6)
- Ineligible control group (n=6)
- Ineligible population (n=5)
- Protocol (n=2)
- Completed trial with no results posted (n=1)
- Ineligible outcomes (n=1)
- Ineligible intervention (n=4)
- Ineligible reports from included RCTs (n=7)

**Figure S.1.** Preferred Reporting Items for Systematic Reviews and Meta-Analyses (PRISMA) 2020 flowchart of study selection process

**Figure S.2.** Network graphs for safety outcomes. Abbreviations: RR, risk ratio; CI, confidence interval; CA, catheter ablation; AAD, antiarrhythmic drugs; SC, standard care; AE, adverse event; TIA, transient ischemic attack.

**Figure S.3.** Comparison-adjusted funnel plots for efficacy outcomes accompanied by Egger’s test p-value.

**Figure S.4.** Comparison-adjusted funnel plots for safety outcomes accompanied by Egger’s test p-value.

**Figure S.5.** Contribution plot for the outcome of appropriate shock. Abbreviations: CA, catheter ablation; AAD, antiarrhythmic drugs; SC, standard care; RR, risk ratio; CI, confidence interval.

**Figure S.6.** Contribution plot for the outcome of electrical storm. Abbreviations: CA, catheter ablation; AAD, antiarrhythmic drugs; SC, standard care; RR, risk ratio; CI, confidence interval.

**Figure S.7.** Contribution plot for the outcome of VA recurrence. Abbreviations: VA, ventricular arrhythmia. CA, catheter ablation; AAD, antiarrhythmic drugs; SC, standard care; RR, risk ratio; CI, confidence interval.

**Figure S.8.** Contribution plot for the outcome of all-cause mortality. Abbreviations: CA, catheter ablation; AAD, antiarrhythmic drugs; SC, standard care; RR, risk ratio; CI, confidence interval.

**Figure S.9.** Contribution plot for the outcome of CV mortality. Abbreviations: CV, cardiovascular; CA, catheter ablation; AAD, antiarrhythmic drugs; SC, standard care; RR, risk ratio; CI, confidence interval.

**Figure S.10.** Contribution plot for the outcome of HF exacerbation. Abbreviations: HF, heart failure. CA, catheter ablation; AAD, antiarrhythmic drugs; SC, standard care; RR, risk ratio; CI, confidence interval.

**Figure S.11.** Contribution plot for the outcome of undetected VT. Abbreviations: CA, catheter ablation; AAD, antiarrhythmic drugs; SC, standard care; RR, risk ratio; CI, confidence interval.

**Figure S.12.** Contribution plot for the outcome of cardiac hospitalization. Abbreviations: CA, catheter ablation; AAD, antiarrhythmic drugs; SC, standard care; RR, risk ratio; CI, confidence interval.

**Figure S.13.** Contribution plot for the outcome of VA hospitalization. Abbreviations: VA, ventricular arrhythmia. CA, catheter ablation; AAD, antiarrhythmic drugs; SC, standard care; RR, risk ratio; CI, confidence interval.

**Figure S.14.** Contribution plot for the outcome of appropriate ATP. Abbreviations: CA, catheter ablation; AAD, antiarrhythmic drugs; SC, standard care; RR, risk ratio; CI, confidence interval.

**Figure S.15.** Contribution plot for the outcome of any serious AE. Abbreviations: AE, adverse event; CA, catheter ablation; AAD, antiarrhythmic drugs; SC, standard care; RR, risk ratio; CI, confidence interval.

**Figure S.16.** Contribution plot for the outcome of cardiac perforation. Abbreviations: CA, catheter ablation; AAD, antiarrhythmic drugs; SC, standard care; RR, risk ratio; CI, confidence interval.

**Figure S.17.** Contribution plot for the outcome of pericardial effusion (drainage). Abbreviations: CA, catheter ablation; AAD, antiarrhythmic drugs; SC, standard care; RR, risk ratio; CI, confidence interval.

**Figure S.18.** Contribution plot for the outcome of stroke/TIA. Abbreviations: CA, catheter ablation; AAD, antiarrhythmic drugs; SC, standard care; RR, risk ratio; CI, confidence interval.

**Figure S.19.** Contribution plot for the outcome of major bleeding. Abbreviations: CA, catheter ablation; AAD, antiarrhythmic drugs; SC, standard care; RR, risk ratio; CI, confidence interval.

**Figure S.20.** Contribution plot for the outcome of myocardial infarction. Abbreviations: CA, catheter ablation; AAD, antiarrhythmic drugs; SC, standard care; RR, risk ratio; CI, confidence interval.

**Figure S.21.** Contribution plot for the outcome of hypothyroidism. Abbreviations: CA, catheter ablation; AAD, antiarrhythmic drugs; SC, standard care; RR, risk ratio; CI, confidence interval.

**Figure S.22.** Contribution plot for the outcome of hyperhtyroidism. Abbreviations: CA, catheter ablation; AAD, antiarrhythmic drugs; SC, standard care; RR, risk ratio; CI, confidence interval.

**Figure S.23.** Contribution plot for the outcome of pulmonary toxicity. Abbreviations: CA, catheter ablation; AAD, antiarrhythmic drugs; SC, standard care; RR, risk ratio; CI, confidence interval.

**Figure S.24.** Contribution plot for the outcome of liver toxicity. Abbreviations: CA, catheter ablation; AAD, antiarrhythmic drugs; SC, standard care; RR, risk ratio; CI, confidence interval.

**Figure S.25.** Sensitivity analysis including both primary and secondary prevention trials (efficacy outcomes). Abbreviations: CA, catheter ablation; AAD, antiarrhythmic drugs; SC, standard care; RR, risk ratio; CI, confidence interval; VA, ventricular arrhythmia; N/A, not applicable; CV, cardiovascular; HF, heart failure; VT, ventricular tachycardia; ATP, antitachycardia pacing.

**Figure S.26.** Sensitivity analysis including both primary and secondary prevention trials (safety outcomes). Abbreviations: CA, catheter ablation; AAD, antiarrhythmic drugs; SC, standard care; RR, risk ratio; CI, confidence interval; AE, adverse event; TIA, transient ischemic attack.

**Figure S.27.** Sensitivity analysis including only CAD patients (efficacy outcomes). Abbreviations: CAD, coronary artery disease; CA, catheter ablation; AAD, antiarrhythmic drugs; SC, standard care; RR, risk ratio; CI, confidence interval; VA, ventricular arrhythmia; N/A, not applicable; CV, cardiovascular; HF, heart failure; VT, ventricular tachycardia; ATP, antitachycardia pacing.

**Figure S.28.** Sensitivity analysis including only CAD patients (safety outcomes). Abbreviations: CAD, coronary artery disease; CA, catheter ablation; AAD, antiarrhythmic drugs; SC, standard care; RR, risk ratio; CI, confidence interval; AE, adverse event; TIA, transient ischemic attack.

**Figure S.29.** Sensitivity analysis including only studies with n≥100 (efficacy outcomes). Abbreviations: CA, catheter ablation; AAD, antiarrhythmic drugs; SC, standard care; RR, risk ratio; CI, confidence interval; VA, ventricular arrhythmia; N/A, not applicable; CV, cardiovascular; HF, heart failure; VT, ventricular tachycardia; ATP, antitachycardia pacing.

**Figure S.30.** Sensitivity analysis including only studies with n≥100 (safety outcomes). Abbreviations: CA, catheter ablation; AAD, antiarrhythmic drugs; SC, standard care; RR, risk ratio; CI, confidence interval; AE, adverse event; TIA, transient ischemic attack.

**Figure S.31.** Sensitivity analysis including studies that enrolled after 2000 (efficacy outcomes). Abbreviations: CA, catheter ablation; AAD, antiarrhythmic drugs; SC, standard care; RR, risk ratio; CI, confidence interval; VA, ventricular arrhythmia; N/A, not applicable; CV, cardiovascular; HF, heart failure; VT, ventricular tachycardia; ATP, antitachycardia pacing.

**Figure S.32.** Sensitivity analysis including studies that enrolled after 2000 (safety outcomes). Abbreviations: CA, catheter ablation; AAD, antiarrhythmic drugs; SC, standard care; RR, risk ratio; CI, confidence interval; AE, adverse event; TIA, transient ischemic attack.

**Figure S.33.** Sensitivity analysis including studies that enrolled after MADIT-RIT (efficacy outcomes). Abbreviations: CA, catheter ablation; AAD, antiarrhythmic drugs; SC, standard care; RR, risk ratio; CI, confidence interval; VA, ventricular arrhythmia; N/A, not applicable; CV, cardiovascular; HF, heart failure; VT, ventricular tachycardia; ATP, antitachycardia pacing.

**Figure S.34.** Sensitivity analysis including studies that enrolled after MADIT-RIT (safety outcomes). Abbreviations: CA, catheter ablation; AAD, antiarrhythmic drugs; SC, standard care; RR, risk ratio; CI, confidence interval; AE, adverse event; TIA, transient ischemic attack.

## **References of the supplemental material**

[1]	Higgins JPT, Li T, Deeks JJ (editors). Chapter 6: Choosing effect measures and computing estimates of effect. In: Higgins JPT, Thomas J, Chandler J, Cumpston M, Li T, Page MJ, Welch VA (editors). Cochrane Handbook for Systematic Reviews of Interventions v n.d.

[2]	Luo D, Wan X, Liu J, Tong T. Optimally estimating the sample mean from the sample size, median, mid-range, and/or mid-quartile range. Stat Methods Med Res 2018;27:1785–805. https://doi.org/10.1177/0962280216669183.

[3]	Shi J, Luo D, Weng H, Zeng XT, Lin L, Chu H, et al. Optimally estimating the sample standard deviation from the five-number summary. Res Synth Methods 2020;11:641–54. https://doi.org/10.1002/JRSM.1429.

[4]	Redpath CJ, Crean AM, Nery PB, Nair GM, Golian M, Hansom S, et al. Safety and Efficacy of Stereotactic Cardiac Radio-Ablation for Ventricular Tachycardia in Patients at High Risk of Mortality. CJC Open 2025;7:545–54. https://doi.org/10.1016/j.cjco.2025.01.015.

[5]	Nunes-Ferreira A, Brito J, Cortez-Dias N, da Lima da Silva G, Pinto FJ, de Sousa J. Preprocedural imaging guiding ventricular tachycardia ablation in structural heart disease. J Arrhythmia 2025;41. https://doi.org/10.1002/JOA3.13205,.

[6]	Toniolo M, Muser D, Mugnai G, Rebellato L, Daleffe E, Bilato C, et al. Comparison of Oral Procainamide and Mexiletine Treatment of Recurrent and Refractory Ventricular Tachyarrhythmias. J Clin Med 2024;13. https://doi.org/10.3390/JCM13206099,.

[7]	Kotake Y, Huang K, Bennett R, De Silva K, Bhaskaran A, Kanawati J, et al. Efficacy and safety of catheter ablation as first-line therapy for the management of ventricular tachycardia. J Interv Card Electrophysiol 2023;66:1701–11. https://doi.org/10.1007/S10840-023-01483-2,.

[8]	Jiménez Candil J, Castro JC, Hernández J, Fernández-Portales J, Durán M, Durán O, et al. Timing of Ablation and Prognosis of Patients With Electrical Storm and Scar-Related Left Ventricular Dysfunction. Am J Cardiol 2020;136:87–93. https://doi.org/10.1016/j.amjcard.2020.09.010.

[9]	Di Biase L, Romero J, Du X, Mohanty S, Trivedi C, Della Rocca DG, et al. Catheter ablation of ventricular tachycardia in ischemic cardiomyopathy: Impact of concomitant amiodarone therapy on short- and long-term clinical outcomes. Hear Rhythm 2021;18:885–93. https://doi.org/10.1016/j.hrthm.2021.02.010.

[10]	Ibáñez Criado JL, Ortiz M, García-Fernández J, Martínez-Alday JD, Rodríguez Font E, Moreno J, et al. Can ICD Electrograms Help Ventricular Tachycardia Ablation?: Results From the Multicenter Randomized AIDEG-VTA Trial. J Am Coll Cardiol 2025;85:1124–37. https://doi.org/10.1016/j.jacc.2024.10.104.

[11]	Borzov E, Efraim R, Suleiman M, Bar-Deroma R, Billan S, Xie J, et al. Implementing stereotactic arrhythmia radioablation with STOPSTORM.eu consortium support: intermediate results of a prospective Israeli single-institutional trial. Strahlentherapie Und Onkol 2025;201:126–34. https://doi.org/10.1007/S00066-024-02300-Z,.

[12]	Willems S, Tilz RR, Steven D, Kääb S, Wegscheider K, Gellér L, et al. Preventive or Deferred Ablation of Ventricular Tachycardia in Patients with Ischemic Cardiomyopathy and Implantable Defibrillator (BERLIN VT): A Multicenter Randomized Trial. Circulation 2020;141:1057–67. https://doi.org/10.1161/CIRCULATIONAHA.119.043400/FORMAT/EPUB.

[13]	Byrne P, Cullinan J, Smith A, Smith SM. Statins for the primary prevention of cardiovascular disease: an overview of systematic reviews. BMJ Open 2019;9:e023085. https://doi.org/10.1136/bmjopen-2018-023085.

[14]	Reddy VY, Koruth JS, Peichl P, Petru J, Funasako ; Moritoshi, Skoda J, et al. High-Voltage Focal Pulsed Field Ablation to Treat Scar-Related Ventricular Tachycardia: The First-in-Human VCAS Trial. Circulation 2025. https://doi.org/10.1161/CIRCULATIONAHA.125.077025.

[15]	Santangeli P, Zado ES, Supple GE, Haqqani HM, Garcia FC, Tschabrunn CM, et al. Long-Term Outcome with Catheter Ablation of Ventricular Tachycardia in Patients with Arrhythmogenic Right Ventricular Cardiomyopathy. Circ Arrhythmia Electrophysiol 2015;8:1413–21. https://doi.org/10.1161/CIRCEP.115.003562/-/DC1.

[16]	Martinelli-Filho M, Marin-Neto JA, Scanavacca MI, De Paola AAV, Medeiros P de TJ, Owen R, et al. Amiodarone or Implantable Cardioverter-Defibrillator in Chagas Cardiomyopathy: The CHAGASICS Randomized Clinical Trial. JAMA Cardiol 2024;9:1073–81. https://doi.org/10.1001/JAMACARDIO.2024.3169,.

[17]	Tung R, Xue Y, Chen M, Jiang C, Shatz DY, Besser SA, et al. First-Line Catheter Ablation of Monomorphic Ventricular Tachycardia in Cardiomyopathy Concurrent With Defibrillator Implantation: The PAUSE-SCD Randomized Trial. Circulation 2022;145:1839–49. https://doi.org/10.1161/CIRCULATIONAHA.122.060039,.

[18]	Dorian P, Borggrefe M, Al-Khalidi HR, Hohnloser SH, Brum JM, Tatla DS, et al. Placebo-controlled, randomized clinical trial of azimilide for prevention of ventricular tachyarrhythmias in patients with an implantable cardioverter defibrillator. Circulation 2004;110:3646–54. https://doi.org/10.1161/01.CIR.0000149240.98971.A8,.

[19]	Bokhari F, Newman D, Greene M, Korley V, Mangat I, Dorian P. Long-term comparison of the implantable cardioverter defibrillator versus amiodarone: Eleven-year follow-up of a subset of patients in the Canadian Implantable Defibrillator Study (CIDS). Circulation 2004;110:112–6. https://doi.org/10.1161/01.CIR.0000134957.51747.6E,.

[20]	Kühlkamp V, Mewis C, Mermi J, Bosch RF, Seipel L. Suppression of sustained ventricular tachyarrhythmias: A comparison of d,l-sotalol with no antiarrhythmic drug treatment. J Am Coll Cardiol 1999;33:46–52. https://doi.org/10.1016/S0735-1097(98)00521-X.

[21]	Sapp JL, Tang ASL, Parkash R, Stevenson WG, Healey JS, Wells G. A randomized clinical trial of catheter ablation and antiarrhythmic drug therapy for suppression of ventricular tachycardia in ischemic cardiomyopathy: The VANISH2 trial. Am Heart J 2024;274:1–10. https://doi.org/10.1016/j.ahj.2024.04.009.

[22]	Ave MT, Brión M, Fernández BD, Blanco-Verea A, Ocampo TC, Gestal AE, et al. Carvedilol versus flecainide treatment in idiopathic ventricular extrasystoles and tachycardias (CARFLECT IV) trial: Rationale and design. Rev Port Cardiol 2025. https://doi.org/10.1016/J.REPC.2025.07.004.

[23]	Samuel M, Healey JS, Nault I, Sterns LD, Essebag V, Gray C, et al. Ventricular Tachycardia and ICD Therapy Burden With Catheter Ablation Versus Escalated Antiarrhythmic Drug Therapy. JACC Clin Electrophysiol 2023;9:808–21. https://doi.org/10.1016/j.jacep.2023.01.030.

[24]	Baker WL, Kluger J, Coleman CI, White CM. Impact of Magnesium L-Lactate on Occurrence of Ventricular Arrhythmias in Patients with Implantable Cardioverter Defibrillators: A Randomized, Placebo-Controlled Trial. Open Cardiovasc Med J 2015;9:83–8. https://doi.org/10.2174/1874192401509010083,.

[25]	Gojkovic O, Aliot EM, Capucci A, Connolly SJ, Crijns H, Hohnloser SH, et al. Celivarone in patients with an implantable cardioverter-defibrillator: Adjunctive therapy for the reduction of ventricular arrhythmia-triggered implantable cardioverter-defibrillator interventions. Hear Rhythm 2012;9:217-224.e2. https://doi.org/10.1016/j.hrthm.2011.09.073.

[26]	Raitt MH, Connor WE, Morris C, Kron J, Halperin B, Chugh SS, et al. Fish oil supplementation and risk of ventricular tachycardia and ventricular fibrillation in patients with implantable defibrillators: A randomized controlled trial. JAMA 2005;293:2884–91. https://doi.org/10.1001/JAMA.293.23.2884,.

[27]	Singer I, Al-Khalidi H, Niazi I, Tchou P, Simmons T, Henthorn R, et al. Azimilide Decreases Recurrent Ventricular Tachyarrhythmias in Patients with Implantable Cardioverter Defibrillators. J Am Coll Cardiol 2004;43:39–43. https://doi.org/10.1016/j.jacc.2003.07.033.

[28]	Ávila P, Berruezo A, Jiménez-Candil J, Tercedor L, Calvo D, Arribas F, et al. Bayesian analysis of the Substrate Ablation vs. Antiarrhythmic Drug Therapy for Symptomatic Ventricular Tachycardia trial. Europace 2023;25. https://doi.org/10.1093/EUROPACE/EUAD181,.

[29]	Parkash R, Nault I, Rivard L, Gula L, Essebag V, Nery P, et al. Effect of Baseline Antiarrhythmic Drug on Outcomes with Ablation in Ischemic Ventricular Tachycardia: A VANISH Substudy (Ventricular Tachycardia Ablation Versus Escalated Antiarrhythmic Drug Therapy in Ischemic Heart Disease). Circ Arrhythmia Electrophysiol 2018;11. https://doi.org/10.1161/CIRCEP.117.005663,.

[30]	Kuck KH, Tilz RR, Deneke T, Hoffmann BA, Ventura R, Hansen PS, et al. Impact of substrate modification by catheter ablation on implantable cardioverter-defibrillator interventions in patients with unstable ventricular arrhythmias and coronary artery disease: Results from the multicenter randomized controlled SMS (Substrate Modification Study). Circ Arrhythmia Electrophysiol 2017;10. https://doi.org/10.1161/CIRCEP.116.004422/SUPPL_FILE/CIRCAE_CIRCAE-2016-004422-T_SUPP1.PDF.

[31]	Delacrétaz E, Brenner R, Schaumann A, Eckardt L, Willems S, Pitschner HF, et al. Catheter ablation of stable ventricular tachycardia before defibrillator implantation in patients with coronary heart disease (VTACH): An on-treatment analysis. J Cardiovasc Electrophysiol 2013;24:525–9. https://doi.org/10.1111/JCE.12073,.

[32]	Tung R, Josephson ME, Reddy V, Reynolds MR. Influence of clinical and procedural predictors on ventricular tachycardia ablation outcomes: An analysis from the substrate mapping and ablation in sinus rhythm to halt ventricular tachycardia trial (SMASH-VT). J Cardiovasc Electrophysiol 2010;21:799–803. https://doi.org/10.1111/J.1540-8167.2009.01705.X,.

[33]	Nery PB, Wells GA, Tang ASL, Parkash R, Stevenson W, Healey JS, et al. Catheter Ablation versus Sotalol or Amiodarone for Ventricular Tachycardia: A Substudy of the VANISH2 Trial. JACC 2025. https://doi.org/10.1016/J.JACC.2025.09.1595.

[34]	Samuel M, Parkash R, Healey JS, Nault I, Blier L, Gray C, et al. Ventricular Tachycardia Cycle Length and Response to Rhythm Control Therapy: Insights From the VANISH Trial. JACC Clin Electrophysiol 2025. https://doi.org/10.1016/J.JACEP.2025.08.025.

[35]	Deyell MW, Doucette S, Parkash R, Nault I, Gula L, Gray C, et al. Ventricular tachycardia characteristics and outcomes with catheter ablation vs. antiarrhythmic therapy: insights from the VANISH trial. Europace 2022;24:1112–8. https://doi.org/10.1093/EUROPACE/EUAB328,.

[36]	Al-Khatib SM, Daubert JP, Anstrom KJ, Daoud EG, Gonzalez M, Saba S, et al. Catheter Ablation for Ventricular Tachycardia in Patients with an Implantable Cardioverter Defibrillator (CALYPSO) Pilot Trial. J Cardiovasc Electrophysiol 2015;26:151–7. https://doi.org/10.1111/JCE.12567.

[37]	Arenal Á, Ávila P, Jiménez-Candil J, Tercedor L, Calvo D, Arribas F, et al. Substrate Ablation vs Antiarrhythmic Drug Therapy for Symptomatic Ventricular Tachycardia. J Am Coll Cardiol 2022;79:1441–53. https://doi.org/10.1016/J.JACC.2022.01.050.

[38]	Della Bella P, Baratto F, Vergara P, Bertocchi P, Santamaria M, Notarstefano P, et al. Does Timing of Ventricular Tachycardia Ablation Affect Prognosis in Patients With an Implantable Cardioverter Defibrillator? Results From the Multicenter Randomized PARTITA Trial. Circulation 2022;145:1829–38. https://doi.org/10.1161/CIRCULATIONAHA.122.059598/SUPPL_FILE/CIRC_CIRCULATIONAHA-2022-059598_SUPP1.PDF.

[39]	Kuck KH, Schaumann A, Eckardt L, Willems S, Ventura R, Delacrétaz E, et al. Catheter ablation of stable ventricular tachycardia before defibrillator implantation in patients with coronary heart disease (VTACH): a multicentre randomised controlled trial. Lancet 2010;375:31–40. https://doi.org/10.1016/S0140-6736(09)61755-4.

[40]	Reddy VY, Reynolds MR, Neuzil P, Richardson AW, Taborsky M, Jongnarangsin K, et al. Prophylactic Catheter Ablation for the Prevention of Defibrillator Therapy. N Engl J Med 2007;357:2657–65. https://doi.org/10.1056/NEJMOA065457/SUPPL_FILE/NEJM_REDDY_2657SA1.PDF.

[41]	Sapp JL, Wells GA, Parkash R, Stevenson WG, Blier L, Sarrazin J-F, et al. Ventricular Tachycardia Ablation versus Escalation of Antiarrhythmic Drugs. N Engl J Med 2016;375:111–21. https://doi.org/10.1056/NEJMOA1513614/SUPPL_FILE/NEJMOA1513614_DISCLOSURES.PDF.

[42]	Žižek D, Mrak M, Jan M, Mežnar AZ, Ivanovski M, Žlahtič T, et al. Impact of preventive substrate catheter ablation on implantable cardioverter-defibrillator interventions in patients with ischaemic cardiomyopathy and infarct-related coronary chronic total occlusion: The PREVENTIVE VT randomized multicentre trial. EP Eur 2024;26:109. https://doi.org/10.1093/EUROPACE/EUAE109.

[43]	Sapp JL, Tang ASL, Parkash R, Stevenson WG, Healey JS, Gula LJ, et al. Catheter Ablation or Antiarrhythmic Drugs for Ventricular Tachycardia. N Engl J Med 2024. https://doi.org/10.1056/NEJMOA2409501/SUPPL_FILE/NEJMOA2409501_DATA-SHARING.PDF.

[44]	Seidl K, Hauer B, Schwick NG, Zahn R, Senges J. Comparison of Metoprolol and Sotalol in preventing ventricular tachyarrhythmias after the implantation of a cardioverter/defibrillator. Am J Cardiol 1998;82:744–8. https://doi.org/10.1016/S0002-9149(98)00478-0.

[45]	Pacifico A, Hohnloser SH, Williams JH, Tao B, Saksena S, Henry PD, et al. Prevention of Implantable-Defibrillator Shocks by Treatment with Sotalol. N Engl J Med 1999;340:1855–62. https://doi.org/10.1056/NEJM199906173402402/ASSET/A1FCA977-871C-4BED-A99C-7DEC75B39368/ASSETS/IMAGES/LARGE/NEJM199906173402402_T5.JPG.

[46]	Kettering K, Mewis C, Dörnberger V, Vonthein R, Bosch RF, Kühlkamp V. Efficacy of Metoprolol and Sotalol in the Prevention of Recurrences of Sustained Ventricular Tachyarrhythmias in Patients with an Implantable Cardioverter Defibrillator. Pacing Clin Electrophysiol 2002;25:1571–6. https://doi.org/10.1046/J.1460-9592.2002.01571.X.

[47]	Connolly SJ, Dorian P, Roberts RS, Gent M, Bailin S, Fain ES, et al. Comparison of β-Blockers, Amiodarone Plus β-Blockers, or Sotalol for Prevention of Shocks From Implantable Cardioverter Defibrillators: The OPTIC Study: A Randomized Trial. JAMA 2006;295:165–71. https://doi.org/10.1001/JAMA.295.2.165.

[48]	Raatikainen P, Mäkynen H, Hartikainen J, Urstad MJ, Konkola L, Sandgaard NCF, et al. Early substrate-based catheter ablation vs. antiarrhythmic drug therapy for ventricular tachyarrhythmias among patients with prior myocardial infarction: the MANTRA-VT randomized trial. EP Eur 2025;27. https://doi.org/10.1093/EUROPACE/EUAF236.
